# Focusing on the interplay between tumor-associated macrophages and tumor microenvironment: from mechanism to intervention

**DOI:** 10.7150/thno.113727

**Published:** 2025-06-20

**Authors:** Hancheng Wu, Jing Li, Ruilin Yao, Jing Liu, Lili Su, Wenjie You

**Affiliations:** 1 Department of Respiratory and Critical Care Medicine, Shandong Provincial Hospital affiliated to Shandong First Medical University, Jinan, Shandong 250021, China.; 2 Department of Respiratory and Critical Care Medicine, Shandong Provincial Hospital, Shandong University, Jinan, Shandong 250021, China.; 3 Shandong Key Laboratory of Infectious Respiratory Disease, Jinan, Shandong 250000, China.; 4 Medical Science and Technology Innovation Center, Shandong First Medical University & Shandong Academy of Medical Sciences, Jinan, Shandong 250000, China.; 5 Department of Respiratory and Critical Care Medicine, Shandong Provincial Public Health Clinical Center, Jinan, Shandong 250013, China.; 6 Departments of Thoracic Surgery and State Key Laboratory of Genetic Engineering, Fudan University Shanghai Cancer Center, Shanghai 200032, China.

**Keywords:** cancer immunotherapy, tumor-associated macrophages, tumor microenvironment, heterogeneity, interaction

## Abstract

Immunotherapy has generated promising outcomes in cancer treatment; however, therapeutic responses are hampered by immunosuppression in the tumor microenvironment (TME). This has resulted in increased study of key immune cells in the TME as therapeutic interventions. Tumor-associated macrophages (TAMs), a major component of infiltrating immune cells in the TME, display high plasticity, largely dependent on cues received from their surroundings. Although significant progress in metabolomics and single-cell omics has unraveled the metabolic and functional heterogeneity of TAMs across several types of cancer, the development of TAM-targeted therapy remains challenging. In the present review, the crosstalk between TAMs and other components in TME, such as tumor cells, immune cells, cancer-associated fibroblasts, and extracellular matrix is highlighted. Additionally, updated insights into the origin, heterogeneity, and metabolic reprogramming of TAMs are discussed, and relevant approaches of targeting TAMs in clinical investigations are summarized. The present review provides a deeper understanding of TAMs within the microenvironment network, aimed at identifying candidate targets to improve cancer immunotherapy.

## Introduction

Cancer immunotherapy, which is designed to counter immune tolerance and induce antitumor immunity, has contributed substantially to cancer treatment in the past decade. Immunotherapeutic approaches involving immune checkpoint blockade (ICB), chimeric antigen receptor (CAR) T-cell engineering therapy, and tumor vaccines have made significant progress in preclinical and clinical studies 1-3. However, the responsiveness of patients to immunotherapy varies significantly across different tumor types and among individuals. Only a small fraction of patients fully respond to immunotherapy, and the factors underlying responses remain largely unknown 4. Therefore, a deeper understanding of the cancer-immunity cycle is of great importance to increase the efficacy of cancer immunotherapy.

The tumor microenvironment (TME) is composed of tumor cells and nonmalignant immune cells, stromal cells, vascular structures, and extracellular components. Certain immune populations serve as antitumor effectors, such as cytotoxic T lymphocytes (CTLs) and natural killer (NK) cells, while other cell types, including regulatory T (Treg) cells, myeloid-derived suppressor cells (MDSCs), and alternatively activated type 2 (M2) tumor-associated macrophages (TAMs), suppress immune-mediated tumor cytotoxicity and facilitate tumor progression 5. Emerging evidence has indicated that the poor efficiency of cancer immunotherapy is tightly associated with the immunosuppressive status driven by immune cell subsets of a myeloid lineage in the TME 6. Among myeloid cells, TAMs, recruited from circulating monocytes or derived from embryonic precursors and bone marrow as tissue-resident macrophages (TRMs), are abundantly present in the TME of most tumors 7. TAMs are a highly plastic and heterogeneous immune cell type. High-throughput techniques, such as single-cell sequencing, have shown a previously unrecognized image of TAM heterogeneity and complexity in multiple tumor types. Novel insights into the metabolic profiles of TAM subpopulations pave the way for attractive methods for TAM metabolic reprogramming 8. The phenotypic and metabolic versatility underlies their interactions with other components in the TME, including tumor cells, infiltrating immune cells, cancer-associated fibroblasts (CAFs), endothelial cells (ECs), adipocytes, extracellular matrix (ECM), *etc*. In general, TAMs fuel cancer malignancy to promote survival, proliferation, angiogenesis, distant spreading, stemness, immunosuppression, and therapeutic response 9. All the above features of TAMs highlight them as attractive therapeutic targets to aid cancer immunotherapy, which have been extensively investigated in preclinical and clinical studies 9. A broad range of strategies have been developed, from targeting the recruitment, heterogeneity, and metabolism of TAMs, depleting and enhancing the phagocytosis and reprogramming of TAMs, to genetically engineered macrophages. There is intense interest in understanding the biology of TAMs, with the ultimate goal of overcoming the limitations of cancer immunotherapy.

In this review, we first describe the origins of TAMs in TME, based on recruitment, differentiation, and polarization. The heterogeneity, diversity, functional profiles, and metabolic characteristics of TAMs are also reviewed in the setting of various tumors. We summarize the interaction and crosstalk between TAMs and surrounding cellular and noncellular components in the TME to comprehensively show how TAMs influence tumorigenesis and therapeutic efficiency. Finally, the relationship between TAMs and current antitumor treatments, as well as the emerging strategies of targeting TAMs as a therapeutic tool, are discussed.

## 1. Origins of TAMs

### 1.1 Recruitment

**Figure 1:**
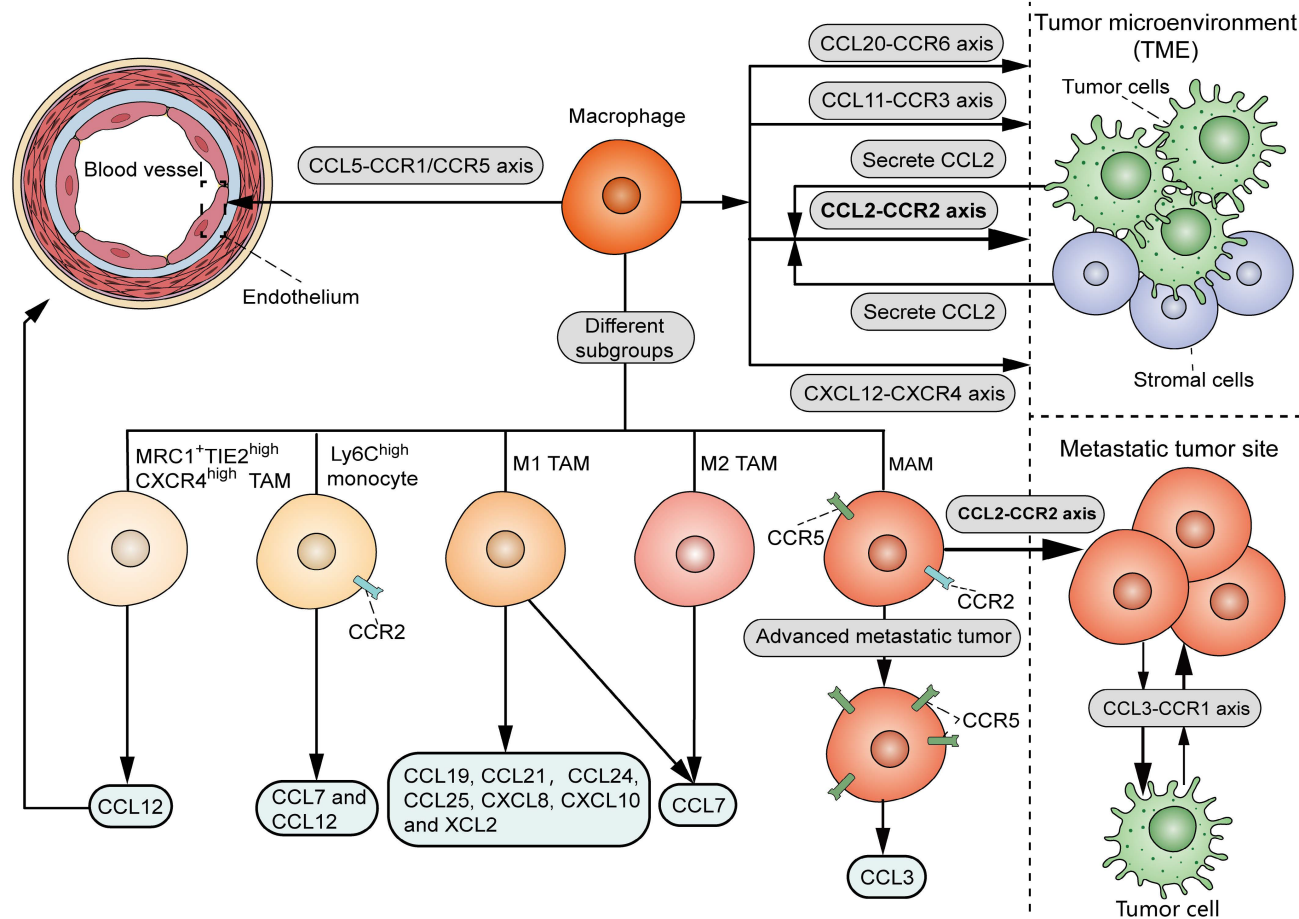
Chemoattractant signals that influence the recruitment of TAMs. TAMs are mainly recruited by the interaction of CCL2, derived from tumor cells and stromal cells within the TME, and CCR2. Other chemoattractant-receptor axis, including CCL20-CCR6, CXCL12-CXCR4 and CCL11-CCR3, also attract macrophages to TME. The interaction of CCL5 with CCR1 and CCR5 promotes monocyte adhesion and immobilization to activated endothelium. As for distinct TAM subsets, M1 TAMs can be recruited by CCL19, CCL21, CCL24, CCL25, CXCL8, CXCL10, XCL2 and CCL7, while M2 TAMs are recruited by CCL7. CCL12 induces chemotaxis of MRC1^+^TIE2^high^CXCR4^high^ TAM subset to the perivascular area. CCL12 and CCL7 recruit inflammatory Ly6C^high^ monocytes via interaction with their co-receptor CCR2. As for different processes in TAM recruitment, while CCL2-CCR2 axis induces the recruitment of MAMs, CCL3-CCR1 axis promotes MAM-cancer cell interaction and MAM retention at the site of metastasis. In advanced stage of metastasis, MAMs migrate to the metastatic site via the CCL3-CCR5 axis.

As shown in **Figure 1**, there are various chemoattractants, including chemokines and growth factors, that influence the recruitment of circulating monocytes into neoplastic tissues. It is widely accepted that the CCL2-CCR2 signal serves as a key determinant for monocyte and TAM recruitment. Bottazzi *et al.* showed that TAMs are recruited by tumor cell-derived chemotactic factors, which were later identified to be CCL2 10. Accordingly, targeting CCL2 with neutralizing antibody *in vivo* or shRNA in tumor cells significantly reduced the infiltration of TAMs in mouse renal cell carcinoma xenografts 11. Notably, CCL2 can originate from other stromal cells within the TME, in addition to tumor cells. Immunohistochemical analysis showed a positive correlation between stromal CCL2 expression and the number of TAMs in human breast cancer samples 12. Consistently, deletion of CCL2 in the host, rather than in tumor cells, significantly decreased TAM infiltration, angiogenesis, and lung metastasis in mouse orthotopical 4T1 tumor models 13. Despite the dominant role of the CCL2-CCR2 axis in TAM recruitment, CCL2 deletion resulted in an approximately 60% TAM reduction in mouse models of endometrial cancer 14, suggesting the involvement of additional chemoattractant signals to attract TAMs. Another study showed that the interaction of CCL5 with CCR1 and CCR5 promotes monocyte adhesion and immobilization to activated endothelium 15, in line with the chemotactic activity of CCL5 in the recruitment of pro-metastatic TAMs in triple-negative breast cancer (TNBC) 16. It was demonstrated that the blockade of CCL20-CCR6 interaction in mouse breast cancer models significantly inhibited TAM recruitment in tumors 17. In mouse models of glioma, TAMs were recruited to tumors when treated with radiation in part through the interaction between CXCL12 (stromal cell-derived factor-1, SDF-1) and its receptor CXCR4 18. Hypoxia induced the production of CCL11 in breast cancer cells, which recruited TAMs to hypoxic regions possibly via interacting with CCR3 19.

Interestingly, it was found that different chemokines attract distinct TAM subsets, which provides a theoretical foundation for isolating specific subsets of TAMs from the TME (**Figure 1**). Xuan *et al.* screened chemokines that differentially recruit classically activated type 1 (M1) or M2 TAMs 20. Their data showed that CCL19, CCL21, CCL24, CCL25, CXCL8, CXCL10 and XCL2 selectively recruited M1 macrophages, while CCL7 induced chemotaxis of both M1 and M2 macrophages. In mouse LLC tumor models, chemotherapy induced CCL12 production, which was specifically chemotactic for the MRC1^+^TIE2^high^CXCR4^high^ TAM subset to the perivascular area 21. Another study showed that CCL12 and CCL7 could recruit inflammatory Ly6C^high^ monocytes via interaction with their co-receptor CCR2 22. It should be noted that different chemokine signals may regulate a specific process involved in TAM recruitment. In murine breast cancer models, the CCL2-CCR2 axis played a vital role in the recruitment of metastasis-associated macrophage (MAM) in metastatic tumors, whereas the CCL3-CCR1 signals specifically promoted MAM-cancer cell interaction and subsequent MAM retention at the site of metastasis 23. Compared to the early MAM accumulation in pulmonary metastasis in a mouse renal tumor model, MAMs increased CCR5 expression in the late stage of metastasis, and migrated to the metastatic site via involvement of the CCL3-CCR5 axis 24. Therefore, targeting TAMs by interfering with certain chemoattractant-receptor interactions may serve as a precise therapeutic approach for cancer management.

### 1.2 Differentiation

**Figure 2:**
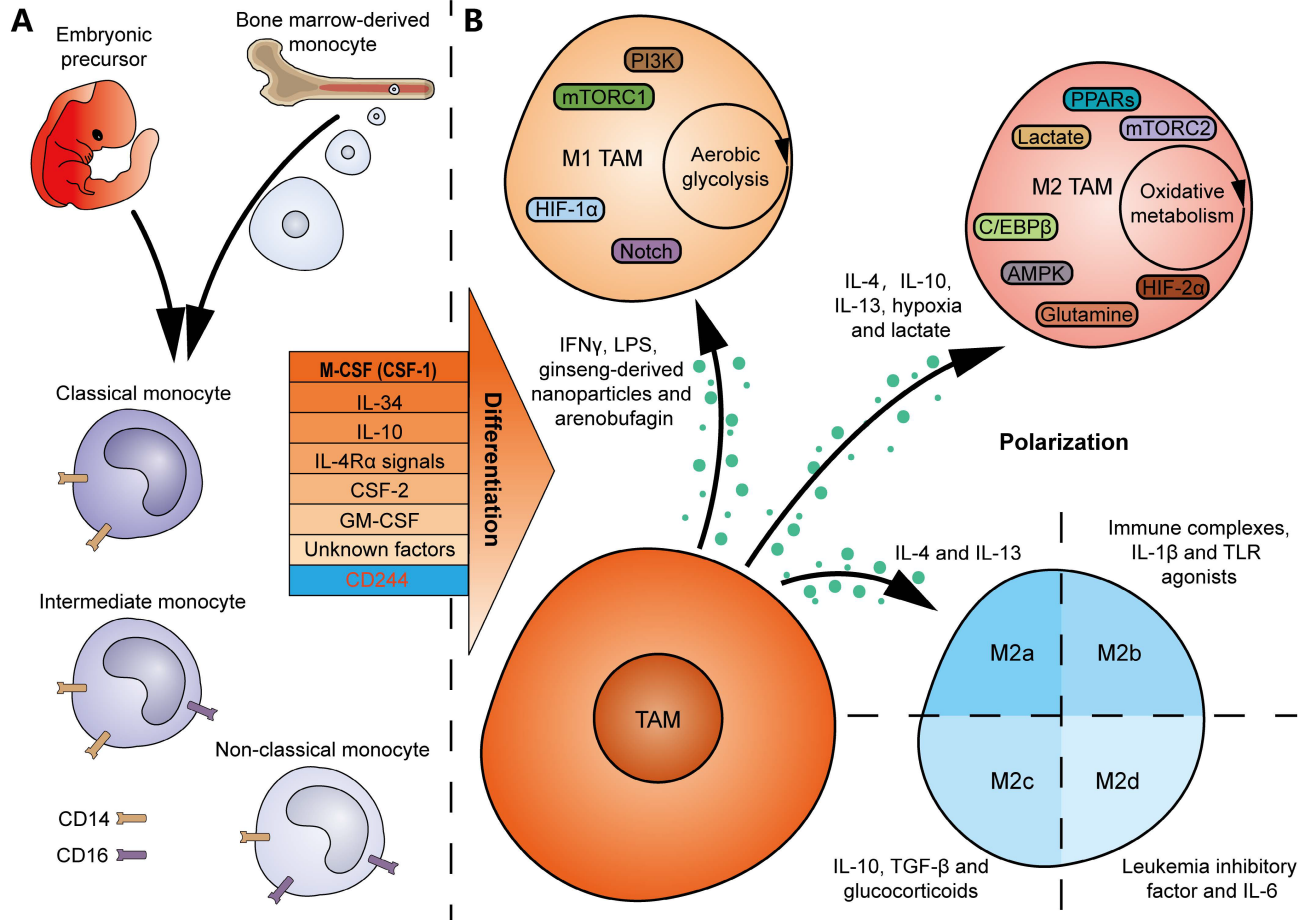
Factors that drive TAM differentiation and polarization. **(A)** TAMs can originate either from embryonic precursors in yolk sac and fetal liver, or from bone marrow-derived monocytes. Many factors, including CSF-1, IL-34, IL-10, IL-4Rα signals, CSF-2, GM-CSF and unknown factors, promote monocyte-to-macrophage differentiation. In contrast, CD244 inhibits the differentiation of anti-tumor macrophages. Human monocytes can be divided into the classical CD14^++^CD16^-^, intermediate CD14^++^CD16^+^, and non-classical CD14^+^CD16^++^ monocytes. **(B)** IFN-γ, LPS, ginseng-derived nanoparticles and arenobufagin drive M1 TAM polarization, metabolically characterized by aerobic glycolysis, whereas IL-4, IL-10, IL-13, hypoxia and lactate drive M2 TAM polarization, characterized by oxidative metabolism. Intrinsic signaling pathways, including PI3K, mTORC1, HIF-1α and Notch signals, are involved in M1 TAM polarization, whereas mTORC2, HIF-2α, AMPK, PPARs, glutamine, lactate and C/EBPβ signals are involved in M2 TAM polarization. Moreover, M2 TAMs can be divided into 4 subgroups: M2a macrophages induced by IL-4 and IL-13, M2b macrophages induced by immune complexes, IL-1β and TLR agonists, M2c macrophages induced by IL-10, TGF-β and glucocorticoids, and M2d macrophages induced by leukemia inhibitory factor and IL-6.

Bone marrow-derived monocytes are considered the origins of multiple TRMs. And TRM-derived TAMs are abundantly present in the TME of several tumor types 25. Many colony-stimulating factors contribute to monocyte-to-macrophage differentiation *in vitro* and *in vivo* (**Figure 2A**). A null mutation in colony-stimulating factor 1 (CSF1) resulted in defective TRMs in mice, suggesting CSF1 as a major regulator of macrophage differentiation 26. IL-34, secreted by keratinocytes in skin epidermis and neurons in central nervous system, regulates the development of local Langerhans cells and microglia 27. Notably, deficiency in CSF1R, the receptor gene for CSF1 and IL-34, led to a greater decline both in the number and diversity of macrophages in mice 28, indicating that macrophages are primarily regulated by the CSF1R signaling. In addition, IL-10 is an inducer of decidual macrophages by promoting oxidative phosphorylation (OXPHOS) in bone marrow-derived monocytes 29. IL-4 receptor α (IL-4Rα) signals in T cells, which is activated following pleural-dwelling nematode infection in C57BL/6 mice, enables the differentiation of resident large-cavity macrophages 30. Dong *et al.* characterized the necessity of CSF-2 in alveolar CD44^+^ macrophage development and maintenance 31. GM-CSF, secreted by intestinal PDGFRA^+^CD142^-/low^ fibroblasts in response to inflammation in inflammatory bowel disease, promotes the transition of monocytes to local CCR2^+^CD206^+^ macrophages 32. Nevertheless, the differentiation of macrophages may depend on unknown growth factors or cytokines, since mice deficient in G-CSF, GM-CSF, and M-CSF still generate macrophages when challenged with sterile peritonitis 33. The above findings suggest that tissue-specific factors or microenvironmental cues should enable local differentiation of macrophages. In contrast, deletion of CD244 in monocytes using Cre-Lox recombination in mice resulted in a higher infiltration of anti-tumor Ly6C^low^ macrophages, demonstrating an inhibitory role for CD244 in anti-tumor macrophage generation 34.

Human monocytes can be classified as follows: (1) classical CD14^++^CD16^-^ monocytes, with proinflammatory and antimicrobial roles; (2) intermediate CD14^++^CD16^+^ monocytes, with proinflammatory roles; and (3) non-classical CD14^+^CD16^++^ monocytes, with patrolling and antiviral roles (**Figure 2A**). An investigation of ovarian cancer revealed a positive correlation between the proportion of intermediate monocytes in peripheral blood and TAMs exhibiting a CCR2^high^CD163^high^CD206^high^CD86^low^ profile in TME 35. Meanwhile, another study on B-cell acute lymphoblastic leukemia suggested that non-classical monocytes in peripheral blood are likely to be the primary source of TAMs in TME 36.

With the development of modern lineage tracing techniques, our understanding of the origins of macrophages has been determined. Several TAMs are derived from TRMs that originate from embryonic precursors in the yolk sac or fetal liver, rather than bone marrow-derived monocytes (**Figure 2A**) 37. Zhu *et al.* characterized the sources of TAMs in mouse pancreatic ductal adenocarcinoma (PDAC) models, showing that pancreas-resident macrophages can originate from embryonic development and increase by *in situ* proliferation during tumor progression 38. The dual origins are believed to determine the heterogeneity of TAM functions: while monocyte-derived TAMs contribute to antigen presentation in immune reactions, embryo-derived TAMs may play a more potent role in the production of the ECM in tumors 38.

### 1.3 Polarization

The phenotype of macrophages is plastic and dynamic, which depends on environmental cues (**Figure 2B**) 39. For example, TAMs can polarize towards an M1 phenotype in response to IFN-γ and lipopolysaccharide (LPS) stimulation, while IL-4, IL-10, and IL-13 are classical cytokines that drive TAM polarization towards an M2 state 39, 40. It should be noted that non-cytokine factors also contribute to TAM polarization, for example, hypoxia and lactate, which drive M2 polarization 39. Additionally, traditional Chinese medicines, such as ginseng-derived nanoparticles and arenobufagin, have been found to promote the polarization of M1 TAMs, thereby achieving anti-tumor effects 41, 42. The intrinsic signaling pathways that influence M1 TAM polarization include PI3K, mTORC1, hypoxia inducible factor-1α (HIF-1α), and the Notch signaling pathway 40. In contrast, the pathways involved in M2 TAM polarization are composed of mTORC2, HIF-2α, AMPK, PPARs, glutamine, lactate, and the C/EBPβ signaling pathway 40. Moreover, metabolic events may play a crucial role in determining their polarization. Typically, M1 macrophages predominantly rely on aerobic glycolysis, whereas M2 macrophages are more dependent on oxidative metabolism, providing potential opportunities to induce reprogramming of TAMs in tumors 43. Furthermore, the M2 macrophages are divided into 4 subgroups, characterized by different inducers (M2a macrophages induced by IL-4 and IL-13, M2b macrophages induced by immune complexes, IL-1β and Toll-like receptor (TLR) agonists, M2c macrophages induced by IL-10, TGF-β and glucocorticoids, and M2d macrophages induced by IL-6 and leukemia inhibitory factor) (**Figure 2B**) 44, 45. These adjustments in classifications better reflect the heterogeneity and diversity of TAMs *in vivo*.

## 2. Phenotypic and functional heterogeneity of TAMs

**Figure 3:**
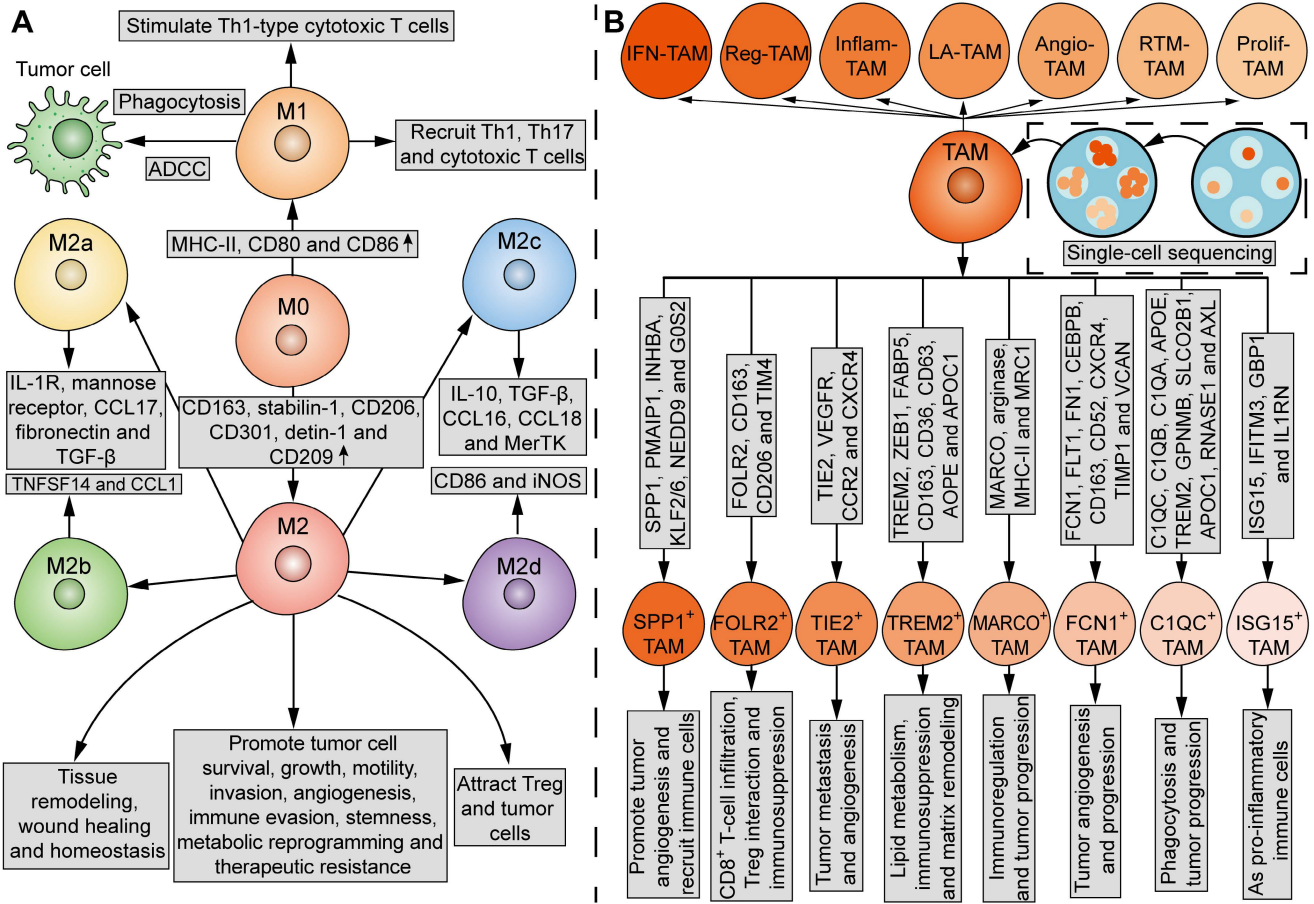
Phenotypic and functional heterogeneity of TAMs. **(A)** Tradditionally, TAMs can be divided into M1 and M2 macrophages, which have different phenotypes and functions. MHC-II, CD80 and CD86 are upregulated in M1 macrophages, while CD163, stabilin-1, CD206, CD301, detin-1 and CD209 are upregulated in M2 macrophages. M1 macrophages can kill tumor cells via direct phagocytosis and ADCC. M1 macrophages also stimulate Th1-type cytotoxic T cells, and recruit Th1, Th17 and cytotoxic T cells. M2 macrophages play an important role in tissue remodeling, wound healing and homeostasis. M2 macrophages promote tumor cell survival, growth, motility, invasion, angiogenesis, immune evasion, stemness, metabolic reprogramming and therapeutic resistance, and attract Treg and tumor cells. Furthermore, M2 macrophages can be divided into four subgroups: M2a, characterized by IL-1R, mannose receptor, CCL17, fibronectin and TGF-β; M2b, characterized by TNFSF14 and CCL1; M2c, characterized by IL-10, TGF-β, CCL16, CCL18 and MerTK; M2d, characterized by CD86 and iNOS. M0, undifferentiated macrophages. **(B)** Current single-cell sequencing have identified seven major subsets of TAMs in tumors: IFN-TAMs, Reg-TAMs, Inflam-TAMs, LA-TAMs, Angio-TAMs, RTM-TAMs and Prolif-TAMs. Moreover, TAMs can be classified into eight subtypes based on the indicated gene signatures and functions: SPP1^+^ TAMs, FOLR2^+^ TAMs, TIE2^+^ TAMs, TREM2^+^ TAMs, MARCO^+^ TAMs, FCN1^+^ TAMs, C1QC^+^ TAMs, and ISG15^+^ TAMs.

TAMs in the TME are not a homogenous cell population. In contrast, subgroups of these TAMs may even have antagonistic functions. The current mainstream classification of TAMs comes from the suggestion put forward by Mills' team in 2000, that TAMs can be divided into M1 and M2 macrophages (**Figure 3A**) 46. In comparison, the undifferentiated macrophages are called M0 macrophages (**Figure 3A**). M1 macrophages upregulate genes involved in antigen processing, presentation, and co-stimulatory signals 39, 47. On one hand, M1 macrophages can kill tumor cells through direct phagocytosis and antibody-dependent cell-mediated cytotoxicity (ADCC). Briefly, when phagocytized by macrophages, tumor cells can be processed into antigen peptides, which induce adaptive antitumor immunity with the assistance of MHC and co-stimulatory molecules 47. On the other hand, M1 macrophages can stimulate Th1-type cytotoxic T cells and recruit Th1, Th17 and cytotoxic T cells 47, 48. M2 macrophages express high levels of CD163, stabilin-1, CD206, CD301, detin-1 and CD209 39, 47. While M2 macrophages play an important role in tissue remodeling, wound healing and homeostasis, certain subsets of M2 macrophages possess tumor-supportive characteristics, such as promoting tumor cell survival, growth, motility, invasion, angiogenesis, immune evasion, stemness, metabolic reprogramming, and therapeutic resistance 49. In addition, M2 macrophages attract Treg and tumor cells 47, 48. However, it is acknowledged that M1 and M2 states do not represent two completely separate subpopulations, but rather two extreme phenotypes that macrophages specifically adopt for the needs of the body. Under certain circumstances, the coexistence of cells with different gene signatures and the presence of a mixture of M1 and M2 macrophages is observed 50. The plasticity of TAMs enables their phenotype and functionality to further lean towards M1 or M2 phenotypes in response to various stimuli from the TME 39. Thus, the M1 and M2 paradigm is inadequate for further analysis of TAM subpopulations.

Mantovani and his colleagues in 2004 proposed to further divide M2 macrophages into M2a (characterized by IL-1R, mannose receptor, CCL17, fibronectin, and TGF-β), M2b (characterized by TNFSF14 and CCL1) and M2c (characterized by IL-10, TGF-β, CCL16, CCL18 and MerTK) macrophages, which exhibit different functions and behaviors (**Figure 3A**) 44. In 2007, Duluc *et al.* discovered a new M2 subpopulation and named it M2d macrophages 45. Compared with M2a-c, M2d macrophages express CD86 and inducible nitric oxide synthetase (iNOS), and exhibit most ovarian TAM phenotypic and functional characteristics, which inhibit T-cell proliferation more effectively 45. Functionally, while M2a and M2b macrophages play immunomodulatory roles, M2c and M2d subpopulations are involved in immune suppression and tissue remodeling 40, 44. In 2015, Igor Malyshev and colleagues proposed the M3 switching phenotype, which is characterized by reprogramming towards the M2 phenotype in response to pro-inflammatory factors or reprogramming towards the M1 phenotype in response to anti-inflammatory factors, inconsistent with traditional views 51. Another study identified a new subgroup of macrophages that can be induced by CXCL4 in atherosclerosis, which was named M4 macrophages 52. However, until now, there has been no literature report on M4 macrophages in the field of tumor biology.

Current single-cell multi-omics approaches have highlighted the heterogeneity of TAMs. A review of single-cell sequencing studies noticed that certain subsets of TAMs are prevalent in almost all cancer types, which fall into seven major classes: interferon-stimulated (IFN-TAMs), immune-modulated (Reg-TAMs), inflammatory cytokine-enriched (Inflam-TAMs), lipid-associated (LA-TAMs), pro-angiogenic (Angio-TAMs), RTM-like (RTM-TAMs), and proliferative TAMs (Prolif-TAMs) (**Figure 3B**) 53. In other studies, TAMs are classified into eight subgroups based on their specific gene signatures and functions: SPP1^+^ TAMs, which are characterized by SPP1, PMAIP1, INHBA, KLF2/6, NEDD9, and G0S2, and act to promote tumor angiogenesis and recruit immune cells, FOLR2^+^ TAMs, which are characterized by FOLR2, CD163, CD206, and TIM4, and are involved in CD8^+^ T-cell infiltration, Treg interaction and immunosuppression, TIE2^+^ TAMs, which are characterized by TIE2, VEGFR, CCR2, and CXCR4, and are involved in tumor metastasis and angiogenesis, TREM2^+^ TAMs, which are characterized by TREM2, ZEB1, FABP5, CD163, CD36, CD63, AOPE, and APOC1, and are involved in lipid metabolism, immunosuppression and matrix remodeling, MARCO^+^ TAMs, which are characterized by MARCO, arginase, MHC-II, and MRC1, and are involved in immunoregulation and tumor progression, FCN1^+^ TAMs, which are characterized by FCN1, FLT1, FN1, CEBPB, CD163, CD52, CXCR4, TIMP1, and VCAN, and are involved in tumor angiogenesis and progression, C1QC^+^ TAMs, which are characterized by C1QC, C1QB, C1QA, APOE, TREM2, GPNMB, SLCO2B1, APOC1, RNASE1, and AXL, and are involved in phagocytosis and tumor progression, and ISG15^+^ TAMs, which are characterized by ISG15, IFITM3, GBP1, and IL1RN, and function as pro-inflammatory immune cells (**Figure 3B**) 54, 55. However, the spectrum of TAM subpopulations may expand beyond current classifications, as the transcriptome signatures of macrophages in tumors are more like continuous and dynamic variables, rather than discrete and fixed phenotypes. Spatial localization and tissue-specific programming may play a major role in determining the phenotypic and functional heterogeneity of TAMs, since the macrophages adopt different functional states in response to local stimulations. By defining the precise TAM subgroups, it may be possible to correlate different TAM subgroups with tumor progression for therapeutic purposes.

## 3. Metabolism of TAMs

Similar to the Warburg effect in tumor cells, cellular metabolism is reprogrammed in TAMs, not only to meet the increased energy demands and biosynthesis, but also to support effector functions, differentiation, and gene expression 40. Increasing evidence suggests a potential correlation between metabolic profiles and the phenotypic and functional characteristics of TAMs 8, 43. Understanding the specific metabolic events in distinct TAM subpopulations is indispensable for metabolic modulation of TAM-associated activity in tumors.

### 3.1 Glucose metabolism

**Figure 4:**
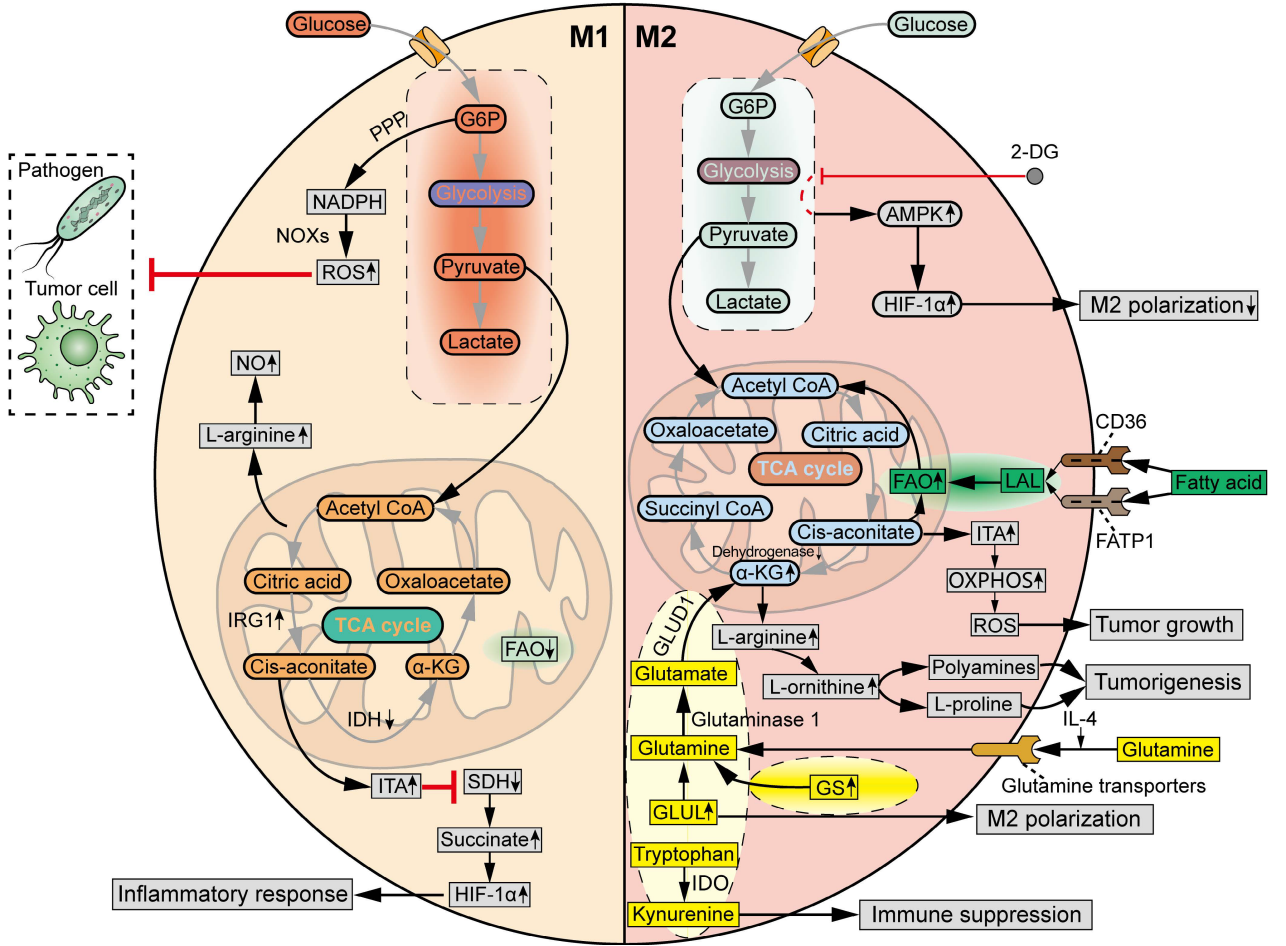
Distinct metabolic profiles of M1 TAMs and M2 TAMs. M1 TAMs are featured by glycolysis, in which G6P is rerouted to PPP to produce NADPH. Then, ROS is generated by NOXs to eliminate pathogens and tumor cells. In M1 macrophage polarization, IDH is downregulated, while IRG1 is upregulated, leading to the production of ITA. ITA inhibits SDH, resulting in the accumulation of succinate to stabilize HIF-1α, which supports the inflammatory response. Meanwhile, L-arginine is increased in M1 TAMs due to interruption of TCA cycle, inducing the synthesis of NO. M2 TAMs are featured by a complete TCA cycle and OXPHOS, fueled by β-oxidation of fatty acids and glutaminolysis. In M2 macrophage polarization, fatty acids can be taken up by CD36 and FATP1, and then lysed by LAL, providing carbon source for FAO to drive TCA cycle and support OXPHOS. Meanwhile, IL-4 increases the uptake of glutamine in macrophages via glutamine transporters. GS and GLUL are upregulated to supply intracellular glutamine for M2 macrophage polarization. Then, glutamine is hydrolysed by glutaminase 1 to produce glutamate, which is transformed into α-KG by GLUD1, in order to fuel TCA cycle and increase FAO and OXPHOS. M2 macrophages can also promote α-KG accumulation via suppressing α-KG dehydrogenase. L-arginine can be converted by Arg-1 into L-ornithine in M2 macrophages. Then, polyamines and L-proline are generated from L-arginine to facilitate tumorigenesis. Tryptophan can be catalyzed by IDO into kynurenine, contributing to immune suppression in M2 macrophages. In response to inflammatory stimuli, glycolytic product ITA is upregulated to potentiate tumor growth by increasing OXPHOS and OXPHOS-driven ROS production. However, glycolysis inhibitor 2-DG can decrease M2 TAM polarization via an AMPK-HIF-1ɑ-dependent pathway. G6P, Glucose-6-phosphate.

Traditionally, the M1 and M2 macrophages display distinct glycometabolic signatures (**Figure 4**). The M1 TAMs rely on glycolysis to fight pathogens and tumor cells, accompanied by a truncated TCA cycle 43. Specifically, the metabolic intermediates of glycolysis, through which glucose is converted to pyruvate and lactate, are rerouted to the oxidative pentose phosphate pathway (PPP) to produce nicotinamide adenine dinucleotide phosphate (NADPH). Then, ROS is generated by NADPH oxidases (NOXs), which are critical for the phagocytic and tumoricidal effects of M1 macrophages 56. The truncated TCA cycle is characterized by the downregulated levels of isocitrate dehydrogenase (IDH) and upregulated levels of aconitate decarboxylase 1 (ACOD1, also known as IRG1) in M1 macrophage polarization, which increase the production of itaconate (ITA) 57. ITA inhibits succinate dehydrogenase (SDH), resulting in the accumulation of succinate to stabilize HIF-1α, which supports the inflammatory response and further strengthens glycolysis 58. Meanwhile, the production of L-arginine is increased in M1 TAMs due to interruption of the TCA cycle, which induces the synthesis of nitric oxide (NO). M2 macrophages exhibit a complete TCA cycle and OXPHOS, with lower glycolytic activity compared to M1 macrophages 43. M2 macrophages tend to utilize β-oxidation of fatty acids and glutaminolysis, rather than glycolysis, to fuel the TCA cycle turnover 59. Furthermore, unlike L-arginine-dependent NO generation in M1 macrophages, the M2 macrophages produce polyamines and L-proline from L-arginine to facilitate tissue homeostasis and tumorigenesis 60.

Even so, the glycometabolism of TAMs exhibits increasingly complex and diverse properties (**Figure 4**). Emerging data indicate that glycolysis is also required for M2 polarization of TAMs. For example, inhibition of glycolysis with 2-Deoxy-d-glucose (2-DG) decreased M2 TAM polarization via an AMPK-HIF-1ɑ-dependent pathway in mouse models 61. Meanwhile, the hypoxic TME induces metabolic shift of TAMs from oxidative metabolism to the glycolytic pathway, which promotes the pro-tumoral M2 phenotype in TAMs and contributes to immune evasion and tumor progression 62. In addition, another glycolytic product, ITA, in response to stimuli such as LPS, TLR, and IFN-γ, was reported to potentiate tumor growth by increasing OXPHOS and OXPHOS-driven ROS production 63.

The heterogeneity of TAMs can be attributed to their ability to modulate key regulators of energy metabolism. MHC-II^high^ TAM subgroup exhibits a hampered TCA cycle. In comparison, low MHC-II expression levels result in higher oxidative and glycolytic metabolism and increased L-arginine metabolism 64. Tim-4^+^ TAMs display higher levels of OXPHOS and arginase-1 (Arg-1), and respond to mitosis to reduce oxidative stress as compared to Tim-4^-^ TAMs 65. Tim-4^+^ TAMs, but not Tim-4^-^ TAMs, promote the peritoneal metastasis of ovarian cancer 65. At present, there remain large gaps in our understanding of the signatures of glucose metabolism in different subgroups of TAMs.

### 3.2 Fatty acid and lipid metabolism

Elevated lipid synthesis in TAMs is tightly associated with tumorigenesis (**Figure 4**). The enhanced fatty acid oxidation (FAO) is one of the most important metabolic features of M2 TAMs. In detail, during M2 macrophage polarization, fatty acids are taken up by the scavenger receptor CD36 and FATP1, and then lysed by lysosomal acid lipase (LAL), to provide a source of carbons for FAO to drive the TCA cycle and support OXPHOS 66. However, other research has suggested that simultaneous induction of fatty acid biosynthesis and FAO may instead direct the polarization of TAMs towards an antitumor phenotype. For example, TLR9 agonism evokes the antitumor potential of TAMs against CD47^+^ cancer cells through activating the FAO and shunting the TCA cycle intermediates to *de novo* lipogenesis 67. Thereafter, it remains to be determined whether and how the coordination of lipid anabolism and catabolism regulates the activities of TAMs within the TME.

High-throughput techniques have depicted the lipid metabolic features in specific subgroups of TAMs. Single-cell and spatially resolved transcriptomics analysis of breast cancer identified two subsets of lipid-associated macrophages (LAM1 and LAM2) 68. LAM1 showed a high expression of FABP5 and abundantly present in invasive cancer areas, while LAM2 showed a high expression of APOE and was primarily located in areas with high stroma, adipocyte, lymphocyte, and high PD-1/PD-L1 staining, indicating that those LAMs are associated with immunosuppressive functions. Another single-cell analysis of early-stage smoking-associated non-small cell lung cancer (NSCLC) patients identified two different immunosuppressive TAM subsets within the TME of NSCLC 54. Specifically, the CCL18^+^ macrophages were characterized by a higher level of fatty acid OXPHOS and exerted immunosuppressive effects by inhibiting the expression of inflammatory factors. A deeper and more comprehensive exploration is required to understand the lipid metabolic reprogramming in those TAM subsets.

### 3.3 Amino acid metabolism

Glutamine, the most abundant circulating amino acid in the blood, is tightly associated with metabolic needs both in tumor cells and M2 macrophages (**Figure 4**). In general, glutamine metabolism is higher in M2 macrophages than in M1 macrophages. IL-4 results in an increased uptake of glutamine in macrophages via glutamine transporters 69. Additionally, glutamine synthetase (GS) is upregulated in glutamine-deprived conditions to replenish the cellular levels of glutamine 70. Glutamate-ammonia ligase (GLUL) was found to be upregulated to maintain the supply of glutamine in M2 TAMs, and inhibition of GLUL decreased glutamine metabolism and resulted in the repolarization of macrophages towards the M1 phenotype 69. Glutamine is hydrolyzed by glutaminase 1 to generate glutamate, which is transformed into α-ketoglutarate (α-KG) by glutamate dehydrogenase 1 (GLUD1), to fuel the TCA cycle and increase FAO and OXPHOS in M2 macrophages 71. Moreover, M2 macrophages can also promote α-KG accumulation by suppressing the enzymatic activity of α-KG dehydrogenase.

The metabolic pathways of other amino acids also exert a significant influence on the phenotypic and functional characteristics of TAMs (**Figure 4**). Tryptophan is catalyzed by the enzyme indoleamine 2,3-dioxygenase (IDO) into kynurenine, also contributing to the immunosuppressive phenotype of TAMs 72. However, further studies are warranted to elucidate the diverse functions of other amino acids in metabolic reprogramming of TAM subpopulations at the cellular level.

## 4. Interactions between TAMs and TME components

The TME is composed of tumor cells, infiltrating immune cells, including TAMs, MDSCs, dendritic cells (DCs), neutrophils, and lymphocytes, stromal cells, such as CAFs, ECs, and the ECM 5. Research on the interactions between certain members of the TME, such as DCs and mast cells, with TAMs is still limited. Therefore, this review only provides an overview of members that have been extensively studied thus far.

### 4.1 Tumor cells

Under steady-state conditions, macrophages are capable of recognizing the “eat me” signal to engulf pathogens, apoptotic cells, or fragments. Conversely, the “do not eat me” signals, such as CD47, PD-L1, and CD24, can inhibit the phagocytic ability of macrophages upon contact. Tumor cells can evade the phagocytic activity of macrophages by increasing the expression of “do not eat me” signals and decreasing the expression of “eat me” signals.

**Figure 5:**
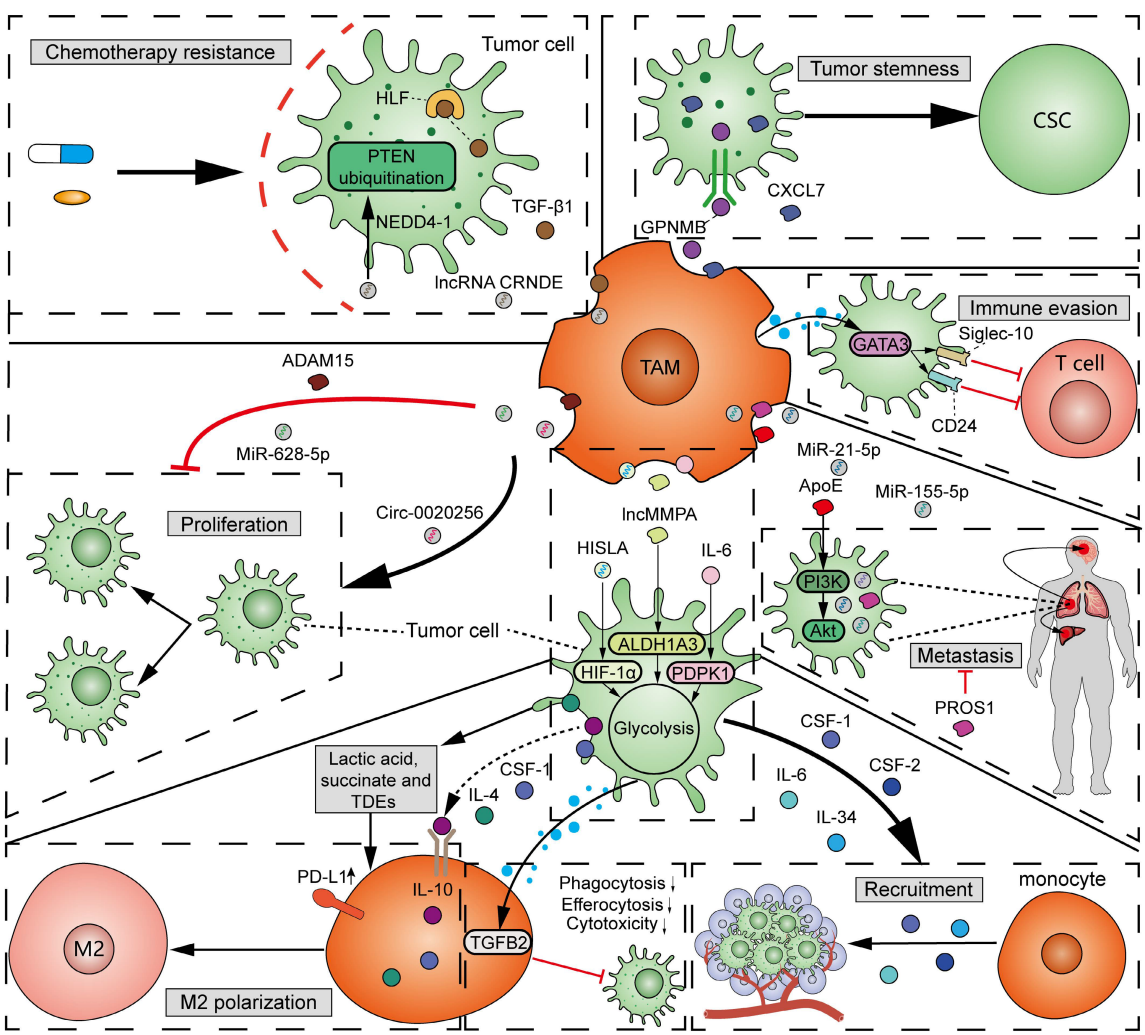
Interactions between TAMs and tumor cells. TAM-derived exosomal circ-0020256 promotes tumor proliferation, while exosomal miR-628-5p and ADAM15 inhibit tumor proliferation. TAM-derived exosomal miR-21-5p and miR-155-5p promote tumor invasion and metastasis, compared to PROS1, which inhibits tumor metastasis. Exosomal ApoE from TAMs promotes tumor migration via activating PI3K-Akt pathway. GATA3 from TAM-derived EVs induces tumor immune evasion via upregulating CD24 and Siglec-10. TAM-derived TGF-β1 and lncRNA CRNDE drive tumor resistance to cisplatin through HLF and NEDD4-1-mediated PTEN ubiquitination, respectively. TAM-secreted GPNMB and CXCL7 restore the stemness of differentiated tumor cells. TAM-derived IL-6, exosomal lncMMPA, and HISLA promote glycolysis in tumor cells via PDPK1, ALDH1A3, and HIF-1α, respectively. Reciprocally, tumor cells recruit circulating monocytes via secreting IL-6, IL-34, CSF-1 and CSF-2. Tumor cell-derived IL-4, IL-10, CSF-1, lactic acid, succinate and TDEs induce M2 TAM polarization. TDEs also induce PD-L1 expression in monocyte-derived TAMs. At last, metastatic TDEs can reduce the phagocytosis, efferocytosis and cytotoxicity of macrophages on tumor cells through TGFB2 signals.

As mentioned above, TAMs, specifically M2 macrophages, participate in tumorigenesis and cancer progression 49. A variety of factors, including protein, metabolites, and non-coding RNAs from TAM-derived extracellular vesicles (EVs), function in the interactions between TAMs and tumor cells (**Figure 5**). (1) Proliferation: Exosomal circ-0020256 from TAMs was found to promote the proliferation of cholangiocarcinoma cells by regulating the miR-432-5p/E2F3 axis pathway 73. However, exosomal miR-628-5p from M1 TAMs was reported to inhibit hepatocellular carcinoma (HCC) proliferation by suppressing the m6A modification of circFUT8 74. In addition, exosomal a disintegrin and metalloproteinase 15 (ADAM15) from TAMs can slow tumor growth and improve survival when co-injected with tumor cells into nude mice 75. (2) Metastasis: By using mass spectrometry, Zheng *et al.* demonstrated that M2 TAM-derived exosomes transfer functional apolipoprotein E (ApoE) to gastric cancer cells, activate PI3K-Akt signaling pathway, and promote tumor migration 76. Exosomal miR-21-5p and miR-155-5p from M2 TAMs boosted the metastasis of colorectal cancer 77. However, PROS1, a protein derived from macrophages, has been identified to exhibit anti-metastatic activities 78. Mechanistically, PROS1 modulated the peripheral inflammation and immune responses, rather than the TAM-related signals within tumor cells, which ultimately decreased tumor metastasis. (3) Immune evasion: Typically, M2 TAMs can facilitate tumor immune evasion by expressing immunosuppressive cytokines and enzymes, and immune checkpoints. Moreover, GATA3 from TAM-derived EVs supported immune evasion of ovarian cancer cells through the CD24/Siglec-10 axis 79. Chiara *et al.* characterized the proteomic and lipidomic profiles of EVs released from mouse TAMs and showed that while TAMs are immunosuppressive, EVs from TAMs show molecular profiles of a Th1/M1 polarization signature and have the potential to stimulate anti-tumor immunity 80. (4) Chemotherapy resistance: TGF-β1 secreted by TAMs can drive cisplatin resistance in TNBC through hepatocyte leukemia factor (HLF) 81. Exosomal lncRNA CRNDE from M2 TAMs promotes the resistance of gastric cancer cells to cisplatin by facilitating neural precursor cell expressed developmentally downregulated protein 4-1 (NEDD4-1)-mediated PTEN ubiquitination 82. (5) Tumor stemness: Cancer stem cells (CSCs) have significant potential for self-renewal and reversible differentiation. TAMs can secrete different products, such as soluble glycoprotein myosone (GPNMB) and CXCL7, to restore the stemness of differentiated tumor cells 83, 84. (6) Tumor metabolism: It was reported that TAM-derived IL-6 promotes 3-phosphoinositide-dependent protein kinase 1 (PDPK1)-mediated phosphoglycerate kinase 1 (PGK1) phosphorylation, promoting glycolysis and malignant behaviors in tumor cells 85. lncMMPA from TAM-derived exosomes interacted with miR-548 to target ALDH1A3, promoting aerobic glycolysis and tumor progression in HCC 86. TAM-derived exosomal HIF-1α-stabilizing long noncoding RNA (HISLA) facilitated the process of aerobic glycolysis and apoptotic resistance in breast cancer cells by activating HIF-1α 87. All the above findings collectively emphasize the important and complex role of TAMs in tumor progression.

It should be noted that the fitness of tumor cells and TAMs sense with each other (**Figure 5**). Tumor cells can induce the recruitment of circulating monocytes into tumor tissues through secreting a variety of cytokines and chemokines, such as IL-6, IL-34, CSF1, and CSF2 88. In addition, tumor-cell-derived cytokines, metabolites, and exosomes affect the polarization and phagocytosis of TAMs, which, in-turn, determine tumor progression and immune evasion. It was reported that tumor cell-derived IL-4, IL-10, CSF-1, lactic acid, and succinate can induce M2 TAM polarization 89, 90. Additionally, several studies have shown that tumor-derived exosomes (TDEs) promote the polarization towards an immunosuppressive M2 phenotype and PD-L1 expression in monocyte-derived TAMs 91. Wolf *et al.* demonstrated that the adoption of exosomes derived from metastatic osteosarcoma cells into mouse alveolar macrophages can reduce the phagocytosis, efferocytosis, and cytotoxicity of macrophages on tumor cells through induction of the TGFB2 signaling pathway (**Figure 5**) 92. The high infiltration of M2 macrophages induced by members of the TME, in addition to tumor cells, is often associated with unfavorable outcomes.

### 4.2 Infiltrating immune cells

#### 4.2.1 T cells

The importance of lymphocytes, which consist of T cells, B cells, and innate lymphoid cells (ILCs) within the TME, is comparable to that of myeloid cells such as TAMs. Among them, T cells are the focus of attention, both in mechanistic and translational studies of immunotherapies, such as ICB and CAR-T cell therapies 1, 2. T cells can be divided by T cell receptor (TCR) subunits into TCRαβ^+^ T cells, which recognize major histocompatibility complex (MHC) class I or class II, and TCRγδ^+^ T cells, which are almost independent of MHC class I and II. In addition, there is another mainstream classification of T cells into CD8^+^ T cells and CD4^+^ T cells according to cell surface CD expression. After recognizing tumor antigens, CD8^+^ T cells can be activated and exert cytotoxic effects by producing perforin, granzyme B, IFN-γ, and FasL/Fas pathways 93. CD4^+^ T cells, which produce cytokines that generate a durable immune response to eliminate pathogens or tumor cells, are primarily referred to as “helper T cells”. Interestingly, another study has shown that CD4^+^ T cells can also exert antitumor activities by direct cytotoxicity 94.

**Figure 6:**
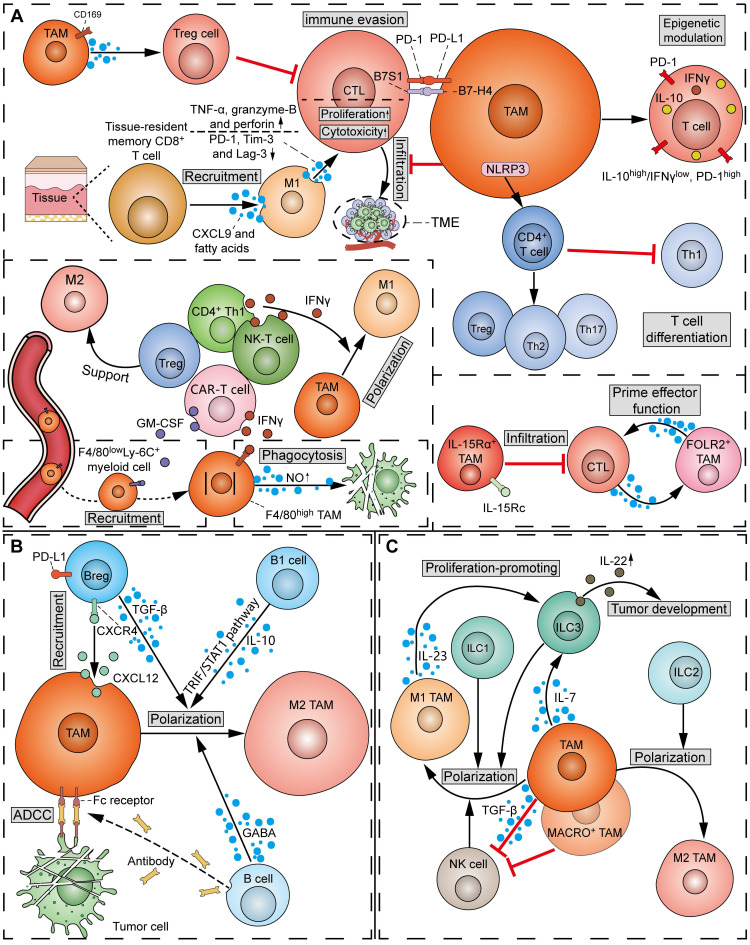
Interactions between TAMs and lymphoid cells in TME. **(A)** Crosstalk of TAMs and T cells: TAMs inhibit the infiltration of CD8^+^ T cells, and suppress CD8^+^ CTLs via the inhibitory B7-H4/B7S1 and PD-L1/PD-1 pathways. CD169^+^ TAMs can recruit Treg cells to suppress CTLs. On the other hand, M1 TAMs induce the recruitment of tissue-resident memory CD8^+^ T cells via CXCL9 and fatty acids. M1 TAMs promote the proliferation and cytotoxicity of CD8^+^ CTLs by upregulating granzyme-B, TNF-α and perforin, and downregulating PD-1, Tim-3 and Lag-3. In addition, TAMs epigenetically modulate tumor-infiltrating T cells into an IL-10^high^/IFN-γ^low^, PD-1^high^ phenotype. TAMs drive the differentiation of Th2, Th17 and Treg cells, and inhibit the polarization of Th1 cells via NLRP3 signals. IL-15Rα^+^ TAMs reduce the infiltration of CD8^+^ T cells by expressing IL-15Rc, while FOLR2^+^ TAMs interact with CTLs and prime effector CD8^+^ T cells. Reciprocally, CD4^+^ Th1 and NK-T cells induce the polarization of M1 TAMs by secreting IFN-γ. CAR-T cells recruit peripheral F4/80^low^Ly-6C^+^ myeloid cells to the TME via secreting GM-CSF, and activate NO production and phagocytosis in F4/80^high^ macrophages via secreting IFN-γ. In contrast, Treg cells support M2 TAMs. **(B)** Crosstalk of TAMs and B cells: CXCL12^+^ TAMs recruit PD-L1^+^ Breg cells via the CXCL12/CXCR4 axis. The M2 polarization of TAMs is promoted by B cell-derived GABA, Breg cell-secreted TGF-β, and B1 cell-derived IL-10 and TRIF/STAT1 pathway. Meanwhile, B cell-derived anti-tumor antibodies bind TAMs' Fc receptors to induce ADCC towards tumor cells. **(C)** Crosstalk of TAMs and ILCs: TAMs induce ILC3 to produce IL-22 via secreting IL-7, thereby promoting tumor development. M1 TAMs promote the proliferation of ILC3s via secreting IL-23. TAMs inhibit the function of NK cells by TGF-β-dependent mechanisms. MACRO^+^ TAMs suppress tumoricidal NK cells. Reciprocally, ILC1s, ILC3s and NK cells drive M1 TAM polarization, while ILC2s drive M2 TAM polarization.

The attitude of TAMs towards T cells can range from “friendly to unfriendly”, which is contingent on the phenotype of both TAMs and T cells. In the process of tumorigenesis, TAMs exert notable influence on the recruitment, activation, proliferation, and effector functions of T cells through the production of chemokines, cytokines, exosomes, or surface immune ligands/receptors (**Figure 6A**). It has been reported that TAMs can suppress CD8^+^ CTLs via the inhibitory B7x (B7-H4/B7S1) molecule in a cell-cell contact manner 95. Moreover, infiltrating CD4^+^ T and CD8^+^ T cells in mouse PDAC models displayed activated IL-10 promoter and repressed T-bet activity (IL-10^high^/IFN-γ^low^, PD-1^high^ phenotype), whereas the infiltrating T cells in TAM-inhibited mouse tumor models exhibited reduced IL-10 and PD-1 levels and activated T-bet promoter activity (IL-10^low^/IFN-γ^high^, PD-1^low^ phenotype), suggesting the epigenetic modulation of infiltrating T cells by TAMs 96. Besides, when CD169^+^ macrophages are adjacent to the tumor, they play an immunosuppressive role by recruiting Treg cells 97. However, M1^hot^ TAMs were found to be positively correlated with CD8^+^ tissue-resident memory T cells, which predicted improved survival in lung cancer 98. M1^hot^ TAMs may induce sustained CD8^+^ tissue-resident memory T-cell recruitment via CXCL9 and essential fatty acids. Using co-culture assays, Zhuang *et al.* showed that the polarized M1 TAMs promoted the proliferation and cytotoxic function of CD8^+^ T by increasing granzyme-B, TNF-α, and perforin expression, and downregulating PD-1, Tim-3, and Lag-3 99. It should be noted that TAMs can influence the differentiation of T-cell subsets. Specifically, NLRP3 signaling in TAMs drives the differentiation of CD4^+^ T cells into tumor-promoting Th2, Th17, and Treg cells, while inhibiting the polarization of Th1 cells 100. The heterogeneity of TAMs in the regulation of T cells has become more apparent. A study on breast cancer showed that IL-15Rα^+^ TAMs reduced tumor infiltration of CD8^+^ T cells by expressing the IL-15/IL-15Rα complex (IL-15Rc) 101. In comparison, a subset of TRMs, defined as FOLR2^+^ TAMs, was found to interact with CD8^+^ T cells and prime effector CD8^+^ T cells in breast cancer 102.

Conversely, T cells also play a crucial role in TAM regulation (**Figure 6A**). Emerging studies report that T-cell subsets capable of releasing IFN-γ, such as CD4^+^ Th1 cells and NK-T cells, can induce M1 polarization of TAMs 103. In a mouse tumor model, the adoptive transfer of T cells expressing a CAR recruited peripheral F4/80^low^Ly-6C^+^ myeloid cells to the TME by secreting GM-CSF, and activated NO production and phagocytosis against tumor cells in F4/80^high^ macrophages by secreting IFN-γ 104. Conversely, Treg cells can facilitate the SREBP1-dependent metabolic fitness, mitochondrial integrity, and survival of M2 TAMs by suppressing CD8^+^ T cell-derived IFN-γ 105.

The relationship between TAMs and T cells in the TME is currently a hot topic of research. However, their interactions are complex, as the phenotypes of both parties and the influence of the surrounding environment should be considered. On one hand, TAMs can directly modulate the activities of anti-tumor T cells. On the other hand, there exists an indirect mechanism through which TAMs determine the immune functions of tumor-infiltrating T cells via their impact on other immune cells. The development of advanced technologies, including single-cell RNA sequencing, spatial transcriptomics and multi-omics, and SpaTial Enhanced REsolution Omics-sequencing (Stereo-seq) 106-108, may assist in understanding the heterogeneity of TAM subtypes, examining cellular variations across different tumor regions, and characterizing intercellular interactions between TAMs and T cells within the TME.

#### 4.2.2 B cells

B cells are predominantly associated with tertiary lymphoid structures (TLSs) in the TME, which can differentiate into plasma cells to produce IgG/IgA in response to tumor-associated antigens 109. Otherwise, B cells can differentiate into regulatory B (Breg) cells to produce immunosuppressive cytokines, including IL-10, IL-35, and TGF-β. The density of TLS and B cell content varies considerably across different tumor types. During tumorigenesis, the effect of B cells has generally been considered as tumor-promoting, either via Breg cell-mediated immunosuppression or via IgG-mediated macrophage activation. On the other hand, though B cells may not have direct effector roles in anti-tumor immunity, a few studies suggested that B cells are involved in anti-tumor immunity by regulating the activation and effector functions of T cells, NK cells, DCs, neutrophils, and TAMs 110.

The crosstalk between myeloid-derived TAMs and B cells has attracted notable attention recently (**Figure 6B**). Lian *et al.* observed co-localization between CXCL12^+^ TAMs and PD-L1^+^ Breg cells in adjacent HCC tissues. Mechanistically, CXCL12^+^ TAMs recruited PD-L1^+^ Breg cells via the CXCL12/CXCR4 axis 111. It is hypothesized that certain glucose-regulated metabolites, for example, cyclic adenosine monophosphate (cAMP), also play an important role in mediating the interaction between TAMs and B cells.

Reciprocally, Zhang *et al.* showed that B cell-derived GABA drives anti-inflammatory polarization of TAMs to weaken the cytotoxic functions of CD8^+^ T cells (**Figure 6B**) 112. As a canonical immunomodulatory factor, TGF-β produced by Breg cells has been shown to skew TAMs towards an immunosuppressive M2 phenotype 113. Another study demonstrated that B1 cells drive TAM polarization towards an M2 phenotype via B1 cell-derived IL-10 and TRIF/STAT1 pathway activation 114. However, the action of B cells on TAMs can be anti-tumorigenic, since the binding of TAMs' Fc receptors with constant regions of anti-tumor antibodies can induce ADCC towards tumor cells 109. Despite the above findings, a landmark discovery is desperately required regarding the functional role of B cells in tumorigenesis, especially with the identification of diverse B-cell clones and subsets through high-throughput technologies. This may be an exciting area of research to uncover the underlying mechanisms governing interactions between tumor-infiltrating B-cell subsets and TAMs in tumor progression.

#### 4.2.3 ILCs

ILCs are derived from the same lymphoid progenitor as T cells, but they differ in structure and function. Currently, the ILC family is classified into five subgroups based on a comprehensive categorization method incorporating cytokines and transcription factors. These subgroups include the helper-like ILC1s, ILC2s, and ILC3s, as well as NK cells and lymphoid tissue-inducer (LTi) cells 115. Notably, both ILC1s and NK cells express high levels of IFN-γ and cytotoxic molecules, which are closely associated with tumor elimination.

In patients with rectal cancer, TAMs can express more IL-7 under the stimulation of *Candida albicans*, and then further induce ILC3 to produce IL-22, ultimately promoting tumor development (**Figure 6C**) 116. Additionally, targeting MARCO, a receptor on the surface of TAM, can alter MACRO^+^ TAM metabolism and enhance the tumor-killing function of NK cells 117. Similar results were obtained in metastatic carcinoma, where TAMs inhibited NK cell function by TGF-β-dependent mechanisms; by contrast, the absence of MAMs promotes the activation, maturation, and number of NK cells, thereby enhancing tumor rejection 118. Instead, IL-23 secreted by M1 macrophages has been shown to promote the proliferation of ILC3s 119.

In turn, ILCs can induce M1/M2 polarization in TAMs, depending on the ILC subpopulation. Specifically, ILC1s, ILC3s, and NK cells can induce the expression of M1 macrophage-related genes, while M2 genes in macrophages can be induced by ILC2s (**Figure 6C**) 120.

#### 4.2.4 MDSCs

MDSCs are a unique type of activated myeloid cells that accumulate in pathological conditions of neutrophil and monocyte accumulation, such as persistent inflammation or tumors. In mice, there are two major subpopulations of MDSC: monocytic (M)-MDSCs (CD11b^+^Ly6G^-^Ly6C^high^) and polymorphonuclear (PMN)-MDSCs (CD11b^+^Ly6G^high^Ly6C^-^). In humans, three subpopulations have been found: major M-MDSCs (CD11b^+^HLA-DR^-/low^CD14^+^CD15^-^), PMN-MDSCs (CD11b^+^HLA-DR^-/low^CD14^-^CD15^+^), and minor early MDSCs (Lin^-^HLA-DR^-^CD33^+^, eMDSCs). Research has indicated that the sole inhibition of TAMs did not lead to a reduction in tumor progression, potentially due to the compensatory emergence of immunosuppressive MDSCs 121.

**Figure 7:**
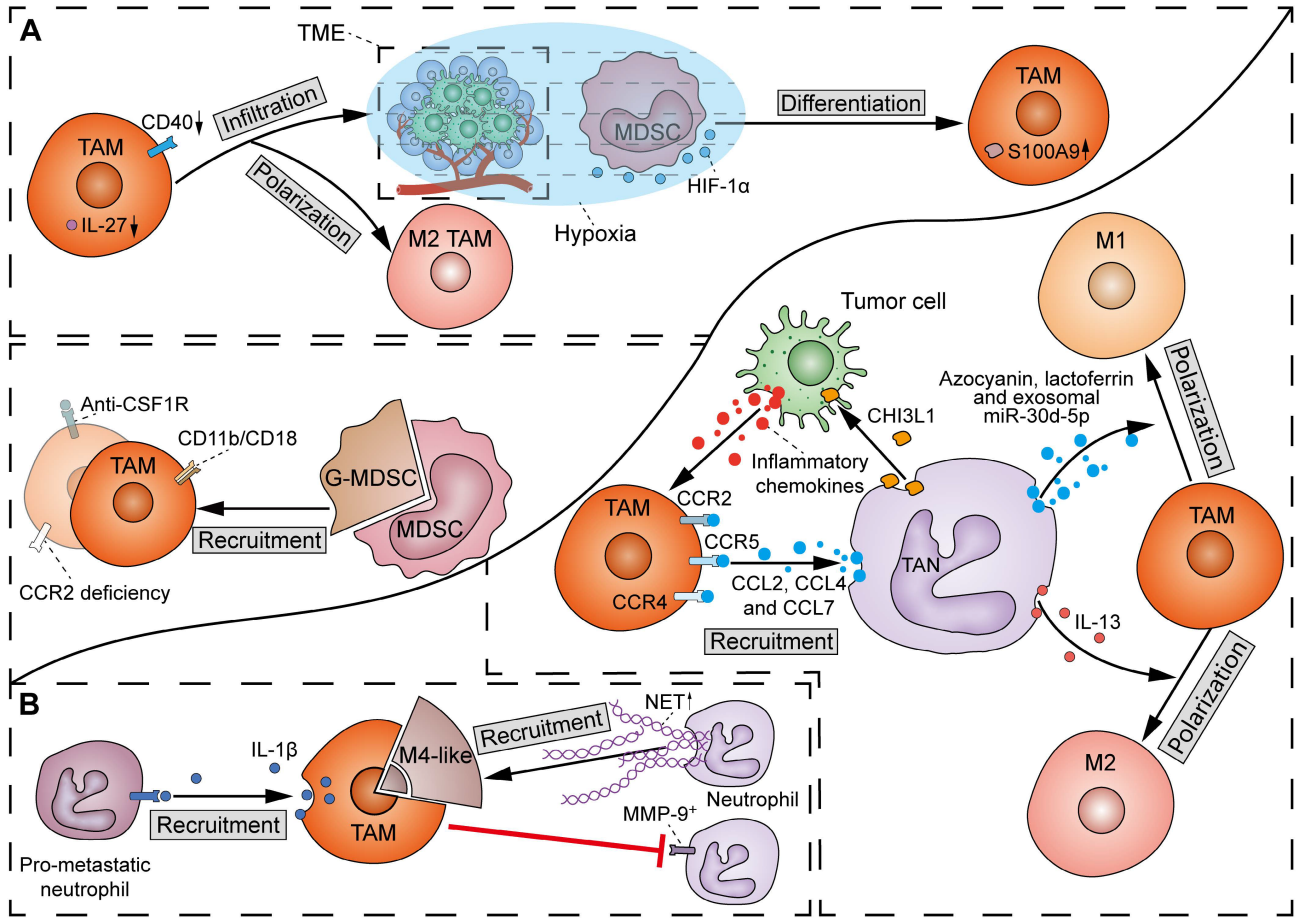
Interactions between TAMs and myeloid cells in the TME. **(A)** Crosstalk of TAMs and MDSCs: TAMs induce the recruitment of MDSCs via expressing CD11b/CD18 integrin heterodimer. However, TAM inhibition by CCR2 deficiency or anti-CSF1R agent also increases G-MDSCs. Reciprocally, MDSCs can induce M2 TAM polarization and infiltration by suppressing CD40/IL-27 signals. Additionally, hypoxia via HIF-1α induces the differentiation of MDSCs into TAMs, with a sustained expression of S100A9. **(B)** Crosstalk of TAMs and TANs: TAMs inhibit the recruitment of MMP-9^+^ neutrophils, except for M4 macrophages, which promote the recruitment of neutrophils and induce the secretion of NETs. On the other hand, TAMs drive the recruitment of pro-metastatic neutrophils to metastatic lesions via producing IL-1β. Reciprocally, TANs promote the recruitment of TAMs through CCL2-CCR2, CCL4-CCR5 and CCL7-CCR4 pathways. Neutrophil-derived CHI3L1 indirectly enhance TAM recruitment by stimulating tumor cells to release inflammatory chemokines. At last, TAN-derived azocyanin, lactoferrin and exosomal miR-30d-5p drive M1 polarization of TAMs, whereas IL-13 initiates M2 TAM polarization.

There has been a scarcity of molecular studies concerning the crosstalk between TAMs and MDSCs (**Figure 7A**). TAMs play a crucial role in facilitating the migration and recruitment of MDSCs to the TME. The M2 metabolic status of TAMs positively correlates with MDSC infiltration. Mechanistically, TAMs express CD11b/CD18 integrin heterodimer (Mac-1; αMβ2), which is one of the essential tools required by MDSCs for their migration and recruitment 122. However, others observed that inhibition of TAM recruitment by CCR2 deficiency or anti-CSF1R agent resulted in a compensatory increase of immunosuppressive G-MDSCs to impair T-cell responses in cholangiocarcinoma 121. Therefore, in most cases, dual inhibition of TAMs and G-MDSCs elicits a more potent effect in potentiating immunotherapy in cancer. Conversely, MDSCs can induce macrophage polarization and infiltration by suppressing CD40/IL-27 signaling to drive melanoma progression and ICB resistance 123. Of note, due to the homogeneity between MDSCs and monocytes, M-MDSCs can differentiate into TAMs. It was found that hypoxia via HIF-1α dramatically induced the differentiation of MDSCs into TAMs within the TME 124. Specifically, TAMs derived from M-MDSCs exhibit potent immunosuppressive properties and M2 polarization tendency due to sustained expression of S100A9 protein.

#### 4.2.5 Neutrophils

Neutrophils are traditionally considered the backbone of primary immune defense. Despite the most abundant leukocytes in human peripheral blood, neutrophils have a short half-life and lifespan, except in the context of inflammatory stimulation, wherein the half-life of neutrophils can be increased by 3.3-fold by 200 U/ml IL-1β 125. The presence of neutrophils in tumors often predicts poor clinical outcomes. For instance, neutrophil extracellular traps (NETs) from dying neutrophils are correlated with tumor progression and metastasis in some studies 126. Nevertheless, neutrophils are highly plastic and heterogeneous cells, some of which exert antitumor functions 127. Neutrophils in the TME, referred to as PMN-MDSCs or tumor-associated neutrophils (TANs), have a simplified bipolar classification: IFN-β signaling pathway induced anti-tumor (N1) TANs and TGF-β signaling pathway induced pro-tumor (N2) TANs. The following studies support the presence of interactions between TAMs and TANs and are also relevant to tumor progression.

There has been emerging evidence regarding the influence of TAMs on TANs, even though the detailed molecular mechanisms are still lacking (**Figure 7B**). A previous study showed that defects in TAM infiltration led to a compensatory recruitment of MMP-9^+^ neutrophils in mouse tumors 128. However, another study found an exception that M4 macrophages derived from M2 macrophages and Kupffer cells promoted the recruitment of neutrophils and induced the secretion of NETs 129. Moreover, Wellenstein *et al*. observed that loss of p53 in breast cancer cells induces the secretion of WNT ligands to increase IL-1β production in TAMs, thereby recruiting pro-metastatic neutrophils to metastatic lesions 130. The above discrepancy may be due to the considerable heterogeneity in intrinsic properties between TAM and TAN subtypes.

TANs can directly promote the recruitment of TAMs by releasing cytokines such as CCL2, CCL4, and CCL7, which bind to surface receptors CCR2, CCR5, and CCR4, respectively (**Figure 7B**) 131. Furthermore, non-enzymatic chitinase-3-like-protein-1 (CHI3L1), a glycoprotein synthesized and released by neutrophils, can also indirectly enhance TAM recruitment by stimulating tumor cells to release inflammatory chemokines 132. TANs can also produce different proteins to influence the polarization of macrophages. Specifically, macrophages can phagocytose TAN-derived azocyanin, lactoferrin, and exosomal miR-30d-5p to promote M1 polarization, or initiate M2 polarization after phagocytosis of IL-13 from the same source 133.

As mentioned previously, TANs can be considered as functional substitutes for TAMs from a certain perspective. Therefore, solely targeting TAMs may not yield effective antitumor effects. It has been demonstrated that simultaneously targeting TAMs and TANs holds significant advantages, such as enhancing chemotherapy response and reducing allergic reactions caused by the use of anti-PD-L1 antibodies. However, targeting TANs is more challenging than targeting TAMs or M-MDSCs due to a lack of tractable targets. The impact of TAMs on the heterogeneity of TANs cannot be overlooked.

### 4.3 CAFs

CAFs, a kind of activated stromal cell capable of secreting collagen, are recognized as a predominant component, as well as the center of communications between different cell types in the TME. CAFs have a variety of precursor cell types, and its molecular markers include but are not limited to α-smooth muscle actin (α-SMA), fibroblast-specific protein 1 (FSP1), fibroblast activation protein (FAP), platelet-derived growth factor receptor-α (PDGFR-α), PDGFR-β, and vimentin. Numerous studies have shown that the interplay between CAFs and TAMs is essential for the formation of immunosuppressive TME.

Experiments on NSCLC models have confirmed that TAMs can transform CAFs, a process known as macrophage-myofibroblast transition (MMT) 134. Additionally, TAMs have the potential to induce myoCAF transformation of CAFs through the CXCL3/CXCR2 axis 135. CCL18 secreted by TAMs induced the conversion of normal breast-resident fibroblasts into CD10^+^GPR77^+^ CAFs, resulting in the enrichment of CSCs and chemoresistance in breast cancer 136.

To date, research has focused on the regulation of CAFs on TAMs. There is ample experimental data showing that CAFs can recruit circulating monocytes into the TME, as well as induce the polarization of TAMs towards M2 phenotype. It was reported that CAFs recruit monocytes and induce STAB1^+^TREM2^high^ LAM via the CXCL12-CXCR4 axis, which supports an immunosuppressive TME in breast cancer 137. In orthotopic and syngeneic colon carcinoma mouse models, IL-6 and GM-CSF from CAFs synergically induce the polarization of monocytes into pre-invasive M2 macrophages 138.

At present, there are far fewer studies on the regulation of CAFs by TAMs than expected. Although CAFs seem to have more regulatory mechanisms for TAMs, crosstalk may exist between pathways in the same direction, although this needs to be experimentally confirmed. From a therapeutic perspective, among these regulatory mechanisms, it may be preferable to select targets that are also essential in other mechanisms. For example, CXCL12 not only promotes M2 polarization of TAMs mediated by CAFs, but also recruits circulating monocytes to TME.

### 4.4 ECs

The process by which new blood vessels sprout from pre-existing blood vessels is called angiogenesis, which not only provides oxygen and nutrients for tumor growth, but is also an important method for tumor metastasis. In particular, angiogenesis plays a vital role in addressing the high metabolic demands of growing tumors. ECs are present in the 80-nm-thick basal lamina located in the innermost layer of blood vessels. Functionally, ECs are not only regulators of vascular tension, but also serve as an important physical barrier and endocrine organ.

TAMs possess the ability to regulate ECs at various levels, including phenotypic and functional aspects. Research by Yang *et al.* indicates that exosomal miR-155-5p and miR-221-5p released by M2 TAMs could be transferred into ECs to further promote angiogenesis 139. Interestingly, in a study on breast cancer, M2 macrophages, but not M1 macrophages, were found to inhibit vascular cellular adhesion molecule-1 (VCAM-1) expression in ECs, which improved vascular integrity 140. Finally, TAMs were reported to induce EC inflammation and promote EC adhesion under hypoxic conditions, which is closely related to inflammation and tumor metastasis 141.

Conversely, ECs in the TME can recruit circulating monocytes, promote monocyte-macrophage transformation, and regulate the polarization of TAMs through different methods. The co-culture of CCR2^+^ monocytes with TNFR2^+^ ECs results in the acquisition of macrophage phenotype 142.

### 4.5 ECM

ECM represents the complex 3D network of structures that surround and support cells within organs and tissues. Furthermore, ECMs also play an important role in regulating cell signaling, function, properties, and morphology 143. In tumors, the four major mechanisms of ECM remodeling, including ECM deposition, post-translational chemical modification, proteolytic degradation, and forced physical remodeling, are disrupted, leading to a tumorigenic ECM 143. CAFs, cancer cells, and certain immune cells (such as TAMs) are the main sources of ECM molecules in the TME. Therefore, exploring the interaction between ECMs and other components may help us to achieve more effective and specialized immunotherapeutic strategies.

Proteoglycan HSPG2 is one of the classic members of several types of tumor ECM and promotes tumor growth by various means. Intriguingly, TAMs have been found to increase the stiffness of the ECM through HSPG2 deposition, which induces immune escape in breast cancer 144. Specifically, TAMs derived from CCR2^+^ monocytes can degrade collagen through receptor-mediated endocytosis via Mrc1 145. TAMs can also indirectly regulate the physical properties and external arrangement of fibers in the ECM through CAFs, depending on the ratio of M1/M2 macrophages. For example, ECM in M2 macrophage-conditioned media had more aligned and thinner fibers than those in mixed M1/M2 macrophage-conditioned media 146.

An earlier study demonstrated that the ECM of different tissues can induce contrasting phenotypes in macrophages, indicating the therapeutic value of targeting ECM molecules 147. The regulatory role of major ECM molecules on TAMs has been gradually elucidated with the advancement of research. For example, collagen internalization can upregulate the expression of Arg-1 and iNOS in TAMs 148. Another fact that demonstrates the potential value of ECM molecules is that, compared with ECM in lean individuals, ECM associated with obesity is more effective in promoting M2 macrophage functions 149.

The process of interaction between TAMs and ECM is characterized by complexity and dynamism. However, it is important to note that ECM is a heterogeneous structure and should not be regarded as a unified entity. It is necessary to divide ECM into different components and explore their respective relationships with TAMs. Targeting TAMs to reshape the tumor-promoting ECM should be a potential antitumor strategy.

## 5. Advances in Targeting TAMs in Cancer Treatment

**Figure 8:**
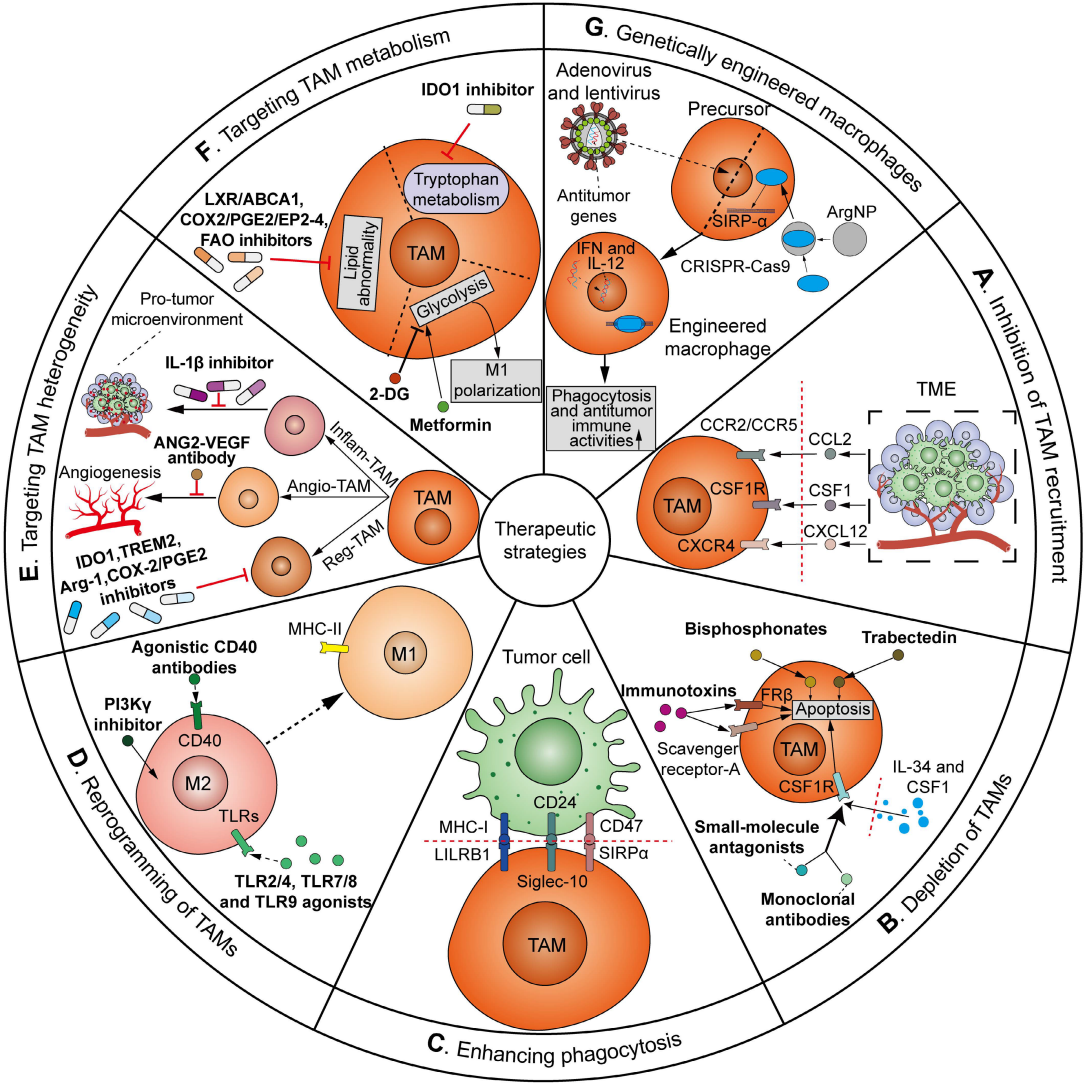
An overview of strategies for TAM-targeting antitumor therapeutics. The ways TAMs have been manipulated can be sorted as follows: **(A)** Inhibition of TAM recruitment. Targeting CCL2-CCR2/CCR5, CSF1-CSF1R, or CXCL12-CXCR4 axis via inhibitors or antibodies can inhibit the recruitment of TAMs to the TME; **(B)** Depletion of TAMs in the TME. CSF1/IL-34-CSF1R blockade via monoclonal antibodies or small-molecule antagonists, bisphosphonates and trabectedin, and targeting scavenger receptor-A or FRβ via immunotoxins can reduce TAM count in TME by inducing apoptosis; **(C)** Enhancing phagocytosis. Blockade of the “do not eat me” signals, *e.g.* SIRPα/CD47 pathway, CD24/Siglec-10 pathway and MHC-I/LILRB1 pathway, can evoke the phagocytic activities of TAMs against tumor cells; **(D)** Reprogramming of TAMs. Agonistic CD40 antibodies, TLR2/4, TLR7/8 and TLR9 agonists, and PI3Kγ inhibitors induce the repolarization of TAMs from M2 towards pro-inflammatory and tumoricidal M1 phenotype; **(E)** Targeting TAM heterogeneity. The heterogeneous TAM subgroups can be targeted by distinct therapeutics. Reg-TAMs are inhibited via IDO1, TREM2, Arg-1 and COX-2/PGE2 inhibitors to blunt immunosuppression. Angio-TAMs are inhibited by ANG2-VEGF antibody to reduce tumor angiogenesis and therapeutic resistance. Inflam-TAMs are inhibited by IL-1β inhibitor to suppress pro-tumor inflammatory and immunosuppressive microenvironment; **(F)** Targeting TAM metabolism. Manipulation of TAMs' metabolism can be achieved via glycolytic modulators 2-DG and metformin to induce M1 TAM polarization, LXR/ABCA1, COX2/PGE2/EP2-4 and FAO inhibitors to treat lipid abnormality, and IDO1 inhibitor to block the tryptophan metabolism; **(G)** Genetically engineered macrophages. Macrophage precursors are genetically engineered through adenoviral or lentiviral transduction of antitumor IFN and IL-12, or ArgNP-mediated CRISPR/Cas9 delivery to knockout SIRP-α gene. Adoptive transfer of genetically engineered macrophages represents a new approach to enhance the phagocytosis and antitumor immune activities of TAMs.

As mentioned above, TAMs play a critical role throughout all stages of cancer, from tumor initiation, metastatic cascade, immune evasion, to cancer therapy resistance, across various cancer types. Importantly, given the prevalence of tumor immunotherapy, there is growing interest in targeting TAMs in clinical trials as adjuvants to current immunotherapies. The means by which TAMs have been manipulated for therapeutic applications can be sorted into the following approaches: (1) inhibition of TAM recruitment; (2) depletion of TAMs in the TME; (3) enhancing phagocytosis; (4) reprogramming of TAMs; (5) targeting TAM heterogeneity; (6) targeting TAM metabolism; and (7) genetically engineered macrophages (**Figure 8**). Many of these strategies have been translated from preclinical models to clinical trials. Herein, we summarize the clinical investigations of agents targeting TAMs in tumors (**Table 1**).

### 5.1 Inhibition of TAM recruitment

To date, the mediators involved in TAM recruitment are diverse and remain incompletely understood. Despite this, a consensus has been reached that the recruitment of circulating monocytes/macrophages is highly dependent on several chemokine signals, some of which may be targeted in antitumor therapy.

*The CCL2-CCR2/CCR5 axis.* The universality of the CCL2-CCR2/CCR5 axis makes it the most attractive target for inhibition of TAM recruitment. Preclinically, targeting CCL2-CCR2 via neutralizing antibodies has yielded encouraging results in delaying tumor progression 186. CCL2-CCR2/CCR5 inhibitors that have been assessed in clinical trials include carlumab (CNTO 888), propagermanium, PF-04136309, CCX872-B, and maraviroc. A Phase 1 trial demonstrated that CNTO 888, a human anti-CCL2 mono-antibody, was well-tolerated with transient free CCL2 suppression and preliminary antitumor activity in solid tumors 150. However, another Phase 1b clinical study of solid tumors found that although no severe adverse events were reported, CNTO 888 did not show long-term CCL2-CCR2 blockade or antitumor effect when combined with chemotherapeutic regimens 151. The reasons may lie in the inadequate clearance of circulating CCL2, since free CCL2 in serum decreased immediately after CNTO 888 treatment but increased with chemotherapy administration. A Phase 1 dose-escalation trial evaluating the effects of propagermanium, an oral organogermanium CCL2 antagonist, in breast cancer patients found that serum IL-6 was downregulated in a dose-dependent manner in the propagermanium-treated patients, implying the promising therapeutic efficacy of propagermanium in cancer angiogenesis and metastasis 152. PF-04136309 is a CCR2 inhibitor, which was assessed in a Phase 1 study of metastatic pancreatic cancer as a combined regimen with gemcitabine and nab-paclitaxel 153. There was a 23.8% ORR in all 21 patients who received PF-04136309; however, the incidence of pulmonary toxicity was relatively high (24%). CCX872-B is a specific CCR2 antagonist. An ongoing Phase 1b trial of pancreatic cancer showed that the overall surviva (OS) rate at 18 months for all patients, including those receiving FOLFIRINOX plus CCX872-B, was 29%, whereas the 18-month OS rate was only 18.6% for FOLFIRINOX treatment 154. Maraviroc, an antagonist of CCR5, has been tested in a Phase 1 clinical trial as an adjuvant to pembrolizumab, which resulted in 5.3% objective response rate (ORR), 2.1-month median progression free survival (PFS), and 9.83-month median OS in refractory mismatch repair proficient colorectal cancer 155. Significantly, another advantage of targeting CCL2-CCR2/CCR5 is the potential for inhibiting the recruitment and function of other immunosuppressive cells, including MDSCs and Treg cells 187, and this may result in a better response.

*The CSF1-CSF1R axis*. The CSF1-CSF1R axis is also critical in the recruitment of TAMs. CSF1R is exclusively expressed by cells of the monocytic lineage, making it a potential target for inhibition of immunosuppressive myeloid-derived cells, especially TAMs. The therapeutic implications of CSF1-CSF1R inhibition in reducing TAM recruitment have been reported in mouse tumor models 188. Antibodies or small-molecule antagonists selectively targeting CSF1-CSF1R have entered Phase 1 and Phase 2 clinical testing, including emactuzumab (RG7155) and lacnotuzumab (MCS110). The humanized anti-CSF1R antibody RG7155 was assessed in a Phase 1 clinical study, which showed that RG7155 effectively blocked the recruitment of immunosuppressive TAMs, but did not result in significant antitumor activities in solid tumors 156. However, another study of diffuse-type tenosynovial giant cell tumor revealed that although facial oedema was found as the most common adverse events, 24 (86%) of all 28 patients treated with RG7155 showed an objective response and two patients achieved a complete response 189, which was predicted to be attributed to the intrinsic feature of diffuse-type tenosynovial giant cell tumors, driven by aberrant CSF1 expression. Roca *et al.* assessed the combinatorial blockade of TAM recruitment with checkpoint immunotherapy in solid tumors 157. They found that RG7155 resulted in a considerable ORR and increased CD8^+^ T-cell infiltration in anti-PD-L1-treated NSCLC patients, suggesting a synergistic antitumor immune response. A Phase 2 clinical study investigated the combinatorial effect of MCS110 with chemotherapy in advanced TNBC 158. However, results showed that while a decrease in CD163^+^ TAMs was observed in lacnotuzumab-treated patients, no additional benefit in terms of increased ORR or improved PFS was observed in the combination treatment group 158. These unexpected results may be attributed to the inadequate selection with regard to high TAM content, since CD163 is a pan-macrophage and monocyte biomarker rather than a specific M2 macrophage biomarker. Therefore, strategies for targeting specific tumorigenic TAM subsets are warranted.

*The CXCL12-CXCR4 axis*. CXCL12-CXCR4 is another signaling axis involved in TAM recruitment and tumor progression. More importantly, since CXCL12 can be induced by conventional cancer treatments, targeting CXCL12-CXCR4 may be a promising adjuvant alongside chemotherapy, radiotherapy, anti-angiogenesis, or immunotherapy by blocking CXCR4^+^ macrophage trafficking. Currently, the majority of drugs targeting CXCL12-CXCR4 in clinical trials are CXCR4 inhibitors, including plerixafor (AMD3100) and BL-8040. The only drug targeting CXCL12 in cancer treatment is olaptesed pegol (NOX-A12). Apart from the mobilization of hematopoietic cells, AMD3100, BL-8040, and NOX-A12 have been evaluated as sensitizing treatment strategies in combination with chemotherapeutic agents or kinase-specific inhibitors in Phase 1 and 2 clinical trials of hematologic tumors 159-161. As for solid tumors, a Phase 1 clinical study found that AMD3100 may exhibit synergistic effects with anti-angiogenic bevacizumab in recurrent high-grade glioma patients 190. Another Phase 2a study showed that BL-8040 increased the clinical benefits of immunotherapy in chemotherapy-resistant PDACs 191.

Although significant achievements have been made in inhibiting the recruitment of TAM-precursors to tumors, the degree and duration of TAM reduction need to be determined and improved. Inhibition of TAM recruitment may lead to compensatory infiltration of TANs and expansion of TRMs, either as monotherapy or in combination therapies, which may compromise the long-term efficacy of this therapeutic approach 128. Notably, the incidence of adverse events likely increases with dose, and this should be considered in clinical use on a per-patient basis.

### 5.2 Depletion of TAMs in TME

Since CSF1R signaling promotes the proliferation, survival, activation, and differentiation of macrophages, CSF1R blockade via monoclonal antibodies or small-molecule antagonists, as listed in **Table 1**, also results in TAM depletion in clinical studies.

Other approaches to reduce TAM counts in the TME employed cytotoxic compounds, including bisphosphonates and trabectedin. Bisphosphonate, a traditional drug for treating cancer bone metastasis and resorption, induces apoptosis after being phagocytosed by TAMs 192. At present, three generations of bisphosphonates have been developed to induce apoptosis by different mechanisms: The first generation etidronate, clodronate, and tiludronate are non-nitrogen-containing bisphosphonates, which can be converted into non-hydrolyzable ATP analogues intracellularly and result in apoptosis. The second-generation tiludronate and third-generation zoledronate are bisphosphonates that induce apoptosis by inhibiting the farnesyl diphosphate (FPP) synthase. Clinically, clodronate and zoledronate have been evaluated as adjuvant agents for treating breast cancer and multiple myeloma 162, 193. Trabectedin is an anticancer agent originally isolated from the Caribbean tunicate *Ecteinascidia turbinata*. Apart from triggering tumor-cell apoptosis, trabectedin was found to selectively induce caspase-8-dependent apoptosis in monocytic cell lineage, including TAMs, through TNF-related apoptosis-inducing ligand (TRAIL) receptors 194. A preclinical study demonstrated that trabectedin administration before anti-PD-1 immunotherapy could alleviate TAM-mediated immunosuppression and thus improve anti-tumor efficacy, which provides a theoretical basis for the combination of trabectedin and immunotherapy 87.

Other approaches to deplete TAMs in tumors have employed immunotoxins that target scavenger receptor-A or folate receptor β (FRβ) expressed on TAMs. Altogether, the major concern about TAM depletion is non-specificity. General depletion of TAMs via the above approaches may lead to the loss of TRMs, which are vital in maintaining homeostasis and bacterial clearance. It is more reasonable and ideal to target specific TAM subsets with high immunosuppressive properties. In support of this notion, depletion of CD163^+^ macrophages resulted in a massive infiltration of activated T cells and tumor reduction in an experimental melanoma model that is insensitive to anti-PD-1 therapy, while the pan-targeting of TAMs did not have therapeutic effects 195.

### 5.3 Enhancing phagocytosis

Macrophages play an essential role via phagocytosis in host defense against pathogens and damaged or aged cells. Normal cells avoid being engulfed by macrophages through the expression of certain molecules, so-called “do not eat me” signals, which can be utilized by tumor cells to evade immune surveillance. There are three regulatory pathways in macrophage phagocytic activities, including the signal-regulated protein α (SIRPα)/CD47 pathway, the CD24/Siglec-10 pathway, and the MHC class I/leukocyte immunoglobulin-like receptor subfamily B member 1 (MHC-I/LILRB1) pathway 196-198. Blockade of the “do not eat me” signals has long been regarded an important therapeutic strategy in evoking the phagocytic function of TAMs to eliminate tumor cells.

CD47 functions as a ligand for SIRPα, which induces a downstream anti-phagocytic cascade in myeloid cells, including TAMs 197. A preclinical study showed the therapeutic efficacy of an anti-CD47 antibody via macrophage phagocytosis in mouse tumor models 199. CD47-SIRPα-targeted agents that have been evaluated in clinical trials include magrolimab (Hu5F9-G4), CC-90002, TTI-621, and BMS-986351. Phase 1 and 2 clinical trials have demonstrated the promising therapeutic efficacy and good tolerability of Hu5F9-G4 in combination with chemotherapeutic agents and anti-CD20 antibody for treating multiple myeloma and non-Hodgkin lymphoma 163, 164. In theory, the bridging between innate and adaptive immunity paves the way for combining phagocytosis-related drugs with ICI-based immunotherapy to elicit more potent antitumor immunity. Drakaki *et al.* evaluated the combination of Hu5F9-G4 with atezolizumab in a Phase 1b/2 open-label, multicenter study of platinum-refractory locally advanced or metastatic urothelial carcinomas 165. Although no improvement in ORR, PFS, or OS in patients treated with atezolizumab plus magrolimab was observed, a trend was observed for increased therapeutic efficacy of atezolizumab plus Hu5F9-G4 in immune-excluded tumors. Maybe the pretreatment selection of CD47 and/or TAM-enriched tumors may improve the efficacy of PD-L1 and CD47 inhibition in this tumor type. Another anti-CD47 antibody, CC-90002, has been evaluated in a Phase 1 study for relapsed/refractory acute myeloid leukemia (AML) and high-risk myelodysplastic syndrome (MDS) 166. However, no objective responses were observed for CC-90002 as a monotherapy. TTI-621 is a SIRPα-IgG1 Fc fusion protein designed to block CD47. A Phase 1 trial showed that TTI-621 was well-tolerated and demonstrated favorable effects both as monotherapy in relapsed/refractory B-cell and T-cell non-Hodgkin lymphomas, and when combined with rituximab in relapsed/refractory B-cell non-Hodgkin lymphomas 167. It is worth noting that CD47 is expressed not only on tumor cells but also on erythrocytes, platelets, and neutrophils. Accordingly, anti-CD47 antibodies inevitably led to the depletion of these normal cells in patients 164. In comparison, approaches to block its counterreceptor SIRPα, expressed on myeloid cells, neutrophils, and microglial cells, are less toxic, but can result in neutropenia and neurotoxicity 200. BMS-986351, a novel anti-SIRPα mAb, is being evaluated in clinical trials for the treatment of advanced solid and hematologic malignancies.

Additional phagocytic checkpoints also regulate the phagocytosis of macrophages. CD24 generates an inhibitory effect on phagocytosis by binding to Siglec-10 198. LILRB1, an inhibitory receptor expressed on macrophages, can interact with MHC-I to inhibit phagocytosis of tumor cells 196. Novel regulators of phagocytosis may be identified via genome-wide overexpression and knockout CRISPR screens in both cancer cells and macrophages, which may serve as promising targets for the development of therapeutic agents to enhance macrophage phagocytosis in the future.

### 5.4 Reprogramming of TAMs

The high plasticity of macrophages provides a rationale for TAM reprogramming in cancer treatment. Emerging studies suggest that re-educating TAMs from tumor-supportive M2 phenotype into antitumor phagocytic and cytotoxic M1 macrophages can be more effective than TAM depletion or recruitment inhibition with regard to killing tumor cells. Additionally, TAM reprogramming is associated with the rebalance of immune infiltrates within the TME. At present, therapeutic approaches primarily focus on the activation of CD40 receptors and TLRs, and the inhibition of phosphatidylinositol-3-kinase-γ (PI3Kγ) pathway.

CD40, a member of the TNF receptor superfamily, is primarily expressed by monocytes, macrophages, DCs, B cells, and epithelial cells 201. Upon binding CD40L, CD40 triggers the upregulation of MHC molecules and the secretion of pro-inflammatory cytokines. Agonistic CD40 antibodies increase TAM infiltration and induce the repolarization of TAMs to favor pro-inflammatory or M1 phenotype in preclinical tumor models 202. Thus, clinical studies of several anti-CD40 agonists, either as monotherapy or in combination with chemotherapy, targeted therapy, and ICB agents in advanced solid tumors have been performed. A Phase 1 dose-escalation study showed that a single intravenous dose of CP-870893, a selective CD40 agonist mAb, was well tolerated and induced objective response and antitumor activity in solid tumors 168. A Phase 1 trial tested whether there were synergetic treatment effects between CP-870893 and chemotherapeutics in advanced solid tumors 169. Both biological responses, such as the depletion of peripheral B cells and the upregulation of immune co-stimulatory molecules, and clinical responses were observed when combining treatment groups. The most common toxicity associated with CP-870893 treatment was cytokine release syndrome (CRS).

TLRs represent one of the major receptor families that polarize macrophages towards a pro-inflammatory and tumoricidal phenotype 203. Preclinical models of cancer have investigated the antitumor immune responses of TLR2/4, TLR7/8, and TLR9 agonists (BCG, 852A, and imiquimod) owing to their properties in TAM modulation. BCG is one of the first TLR2/4 agonists approved for treating bladder cancer patients based on the results of clinical trials 170. A Phase 2 study reported that systemically administered 852A induced systemic immune activation, leading to prolonged disease stabilization in chemotherapy-refractory metastatic melanomas 171. Unfortunately, systemic toxicity, such as fatigue and constitutional symptoms, prevented the use of injections with high levels of TLR ligands in cancer patients. Instead, locally or intratumorally administrated TLR agonists are under evaluation in different tumor models, which have shown the need for achieving a fine balance between effectiveness and toxicity. Weigel *et al.* evaluated subcutaneously delivered 852A in patients with recurrent hematologic malignancies 172. The local 852A treatment resulted in objective responses in 15.4% of hematologic cancer patients. A prospective clinical trial showed that topical imiquimod, a TLR7/8 agonist, was well tolerated and achieved a partial response in 20% of breast cancer skin metastases patients 173. The responders displayed histologic tumor regression and increased tumor lymphocytic infiltration and local cytokine production. It is worth noting that TLR-stimulated TAM is often accompanied by the upregulation of PD-L1 204, which theoretically enables the future combined use of TLR agonists and PD-1/PD-L1 inhibitors in clinical trials.

PI3Kγ is involved in the pro-tumoral activities of TAMs. PI3Kγ inhibition in TAMs induced the expression of MHC-II and pro-inflammatory cytokines and reduced the immunosuppressive molecules, including IL-10 and Arg-1, which contributed to TAM reprogramming. A preclinical study showed that the PI3Kγ inhibitor eganelisib (IPI-549) reprogrammed TAMs and increased CD8^+^ T-cell recruitment, achieving tumor growth inhibition when combined with checkpoint inhibitors 205. Subsequently, a Phase 1/1b MARIO-1 trial demonstrated that IPI-549 achieved antitumor activity when combined with nivolumab in solid tumors, including those who progressed when receiving PD-1/PD-L1 inhibitors 174.

Certain chemotherapeutics, irradiation, or oncolytic virus therapy can induce immunogenic cell death (ICD) of tumor cells, which stimulates antitumor immune responses, and in particular, TAM re-education towards an M1 phenotype, and thus results in additional therapeutic efficiency 206, 207. In this respect, a transcription factor, RORC1/RORγ, orchestrates cancer-driven myelopoiesis, predominantly of TAMs and MDSCs, by promoting C/EBPβ 208. Additionally, several specific inhibitors targeting RORC1/RORγ are under evaluation in preclinical models.

### 5.5 Targeting TAM heterogeneity

New insights into the heterogeneity of TAMs enable the development of novel therapeutics to inhibit TAM-mediated immunosuppression, angiogenesis, and inflammation by targeting immune-related enzymes, ligands, receptors, or signaling transducers. At present, three subgroups of TAMs, including reg-TAMs, angio-TAMs, and inflam-TAMs, are being targeted in clinical trials.

Reg-TAMs express IDO1, TREM2, Arg-1, and the COX-2/PGE2 pathway to induce T-cell exhaustion and Treg infiltration. In a Phase 1a trial, targeting IDO1 enzyme with a small-molecule inhibitor, navoximod (GDC-0919), displayed promising effects in recurrent/advanced solid tumors 175. TREM is an essential immunosuppressive gene in Reg-TAMs. Molgora *et al.* reported that Trem2^-/-^ mice are more resistant to the growth of various cancers and more sensitive to PD-1 blockade therapy than wild-type mice 209. However, targeting TREM2^+^ Reg-TAMs with PY314, a humanized anti-TREM2 mAb, in combination with PD-1 blockade yielded limited anti-tumor effect in patients with checkpoint inhibition-refractory renal cell carcinoma in the setting of a Phase 1b clinical trial 176. Arg-1 represents an immunosuppressive enzyme in myeloid cells and induces depletion of L-arginine, an essential nutrient for T cell and NK cell proliferation. A preclinical study found that CB-1158, a small-molecule Arg-1 inhibitor, shifted the immune landscape towards a pro-inflammatory environment, blunted myeloid cell-mediated immunosuppression, and reduced tumor growth in multiple mouse models of cancer 210. The inflammatory COX-2/PGE2 pathway has been implicated in eliciting immune escape and tumor progression by recruiting and activating Reg-TAMs. In a Phase 2 study, COX-2 inhibitor celecoxib led to a higher pathological complete response rate and an acceptable safety profile when combined with toripalimab as neoadjuvant drugs for mismatch repair-deficient or microsatellite instability-high metastatic colorectal cancer patients 177.

Angio-TAMs are prevalent in the hypoxic core of solid tumors, which facilitate angiogenesis and mediate therapeutic resistance to anti-VEGF agents. ANG-2 confers resistance to anti-VEGF treatment by recruiting angio-TAMs 211. In a preclinical setting, dual blockade of VEGF and ANG2 enhanced the normalization of tumor vasculature and suppressed tumor progression compared with each therapy alone in mouse tumor models 212. A Phase 1 study even showed that when combined with ezabenlimab, BI 836880, a humanized bispecific ANG2-VEGF antibody, had a manageable safety profile with preliminary clinical activity in advanced solid tumors 178. Further clinical trials are warranted to explore the efficiency of targeting angio-TAMs to improve resistance to anti-VEGF or ICI-based treatments.

Inflam-TAMs play an essential role in tumorigenic processes by maintaining a pro-tumor inflammatory and immunosuppressive microenvironment. Numerous inflammatory genes, such as IL-1β, from inflam-TAMs have been evaluated as anti-tumor targets in preclinical investigations 213. A Phase 3 clinical trial evaluated the effect of first-line canakinumab, an IL-1β-blocking antibody, in conjunction with chemotherapy and immunotherapy in advanced NSCLC 179. The addition of canakinumab did not prolong PFS or OS in NSCLC patients. Several other approaches targeting inflam-TAMs are still in preclinical investigations for cancer treatment 214.

### 5.6 Targeting TAM metabolism

There is a growing consensus on the importance of metabolic regulation of immune cells, including TAMs, in reactivating anti-tumor immunity. A list of metabolic intermediates, by-products, and enzymes, generated or activated in the TME in terms of nutrient deprivation, hypoxia, and an acidic environment, underlie the recruitment, activation, expansion, and function of TAMs, which serve as potential targets to reprogram TAMs.

Given that glycolysis and intermediate metabolite lactate are required for the polarization of M2 TAMs, glycolytic inhibitors, such as 2-DG, have been investigated to revert macrophage polarization in a preclinical study 61. A Phase 1 trial showed that 2-DG at 63 mg/kg combined with docetaxel was well-tolerated and resulted in a partial response in one metastatic breast cancer patient and stable disease in eleven solid tumor patients 180. However, since glycolysis is fundamental for the phagocytic and tumoricidal function of M1 TAMs, glycolytic pathway blockade may result in undesirable M1 TAM suppression, which may explain the above limited clinical benefits of 2-DG treatment. Metformin, a mitochondrial respiratory chain complex I inhibitor to promote glucose uptake and glycolysis, has also emerged as a therapeutic candidate in TAM repolarization 215. Preclinically, metformin was found to reeducate M2 TAMs towards an M1 phenotype, which reversed a tumor immunosuppressive microenvironment and synergized with anti-PD-1 immunotherapy in mouse tumor models 216. Mounting evidence from clinical trials has dissected the encouraging antitumor effects of metformin in prostate and endometrial cancer 181, 182. The mechanism of antitumor activities of metformin is partially attributed to tumor immune microenvironment reprogramming, based on the repolarization of macrophages to an antitumoral M1 phenotype and increased infiltration of CD8^+^ T cells and CD20^+^ B cells in metformin-treated mouse esophageal squamous cell carcinoma models 217. However, conflicting data and inconclusive results have also been reported. The addition of anti-diabetic doses of metformin did not improve outcomes in early breast cancer and advanced-stage NSCLC treated with standard therapies 218, 219. Differences in the patient selection, including the diabetic conditions and diets and sensitivity of tumors to energetic stress, constitute major determinants of their responses to metformin and antitumor efficiency. Further trials are required to validate the beneficial effects of metformin in different cancer types.

TAMs display impaired lipid handling, which correlates with the activation of immunosuppressive pathways and the emergence of therapeutic resistance. A preclinical study showed that the cholesterol-lowering simvastatin can induce TAM repolarization from an M2 to M1 phenotype via cholesterol-associated LXR/ABCA1 regulation, resulting in the reversion of EMT-associated resistance to chemotherapy 220. Future clinical studies are required to evaluate cholesterol metabolism modulators in cancer treatment. Additionally, tumor-derived prostaglandin E2 (PGE2) induces the transformation of myeloid cells toward an immunosuppressive phenotype. Targeting of COX2/PGE2/EP2-4 pathway with nonsteroidal and steroidal anti-inflammatory drugs enhanced the efficacy of immune checkpoint inhibitors in mouse tumor models 221. FAO represents another metabolic hallmark in immunosuppressive TAMs. A previous study showed that inhibition of free fatty acid production can repolarize TAMs to a pro-inflammatory phenotype, promoting secretion of tumor-killing cytokines in pancreatic adenocarcinoma tumor models 222. However, another study showed that TLR9 agonist, CpG oligodeoxynucleotides, increased the membrane fluidity of macrophages and enhanced the phagocytosis of tumor cells through promoting intracellular FAO by activating carnitine palmitoyltransferase 1A (CPT1A) and citrate lyase 67.

Abnormal glutamine, arginine, and tryptophan metabolism is intricately associated with the immunosuppressive activities of M2 TAMs, highlighting the possibility of targeting amino acid metabolism as an anti-tumor strategy. Several small-molecule inhibitors targeting IDO1 have been assessed in clinical studies, including epacadostat and indoximod. However, the addition of IDO1 inhibitors to immunotherapy yielded limited antitumor activity in sarcoma and melanoma 183, 184. It is hypothesized that the compensatory expression of other immunosuppressive enzymes, including tryptophan 2,3-dioxygenase (TDO) and IDO2, may limit the effects of IDO1 inhibitors.

Other approaches include the epigenetic modulation and DNA damage repair of TAMs, among other mechanisms*.* A Phase 1 study showed early signs of efficacy of a histone deacetylase (HDAC) inhibitor, tefinostat (CHR-2845), in patients with advanced hematologic malignancies 185. Another preclinical study showed that PARP inhibitors enhanced both anti- and pro-tumor properties of TAMs through glucose and lipid metabolic reprogramming in BRCA-deficient TNBC models 223. Of note, non-specific drugs affecting shared metabolic pathways can impact numerous cellular components within the TME, potentially resulting in unpredictable side-effects and poor effectiveness in cancer treatment. Identification of more specific metabolic transcription factors, pathways, or byproducts involved in TAM reprogramming is required to better strategically manage the various types of cancer.

### 5.7 Genetically engineered macrophages

Advances in cellular engineering methods hold notable potential, including reprogramming macrophages, as a promising anti-tumor strategy. In comparison with adoptive T cells, genetically engineered macrophages infiltrate the TME more efficiently, functioning not only in tumor cell phagocytosis, but also in neo-antigen presentation to tumoricidal immune cells. Macrophage precursors, including circulating monocytes and isolated hematopoietic stem cells (HSC), can be genetically modified by using adenoviral or lentiviral transduction, or gene editing using CRISPR-Cas9 technology 224.

Genetically engineered macrophages were obtained through lentiviral transduction of antitumor genes, such as IL-12, into macrophage precursors to activate antitumor immune responses 225. By using viral protein X (VPX)-containing lentivirus, Klichinsky *et al.* engineered macrophages with an anti-human EGF receptor 2 (HER2) CAR with a CD3ζ cytosolic domain that recognized tumoral HER2 antigen, and this transformed macrophages into a pro-inflammatory phenotype, enhanced antigen-specific phagocytosis, and reduced tumor growth and metastasis in xenograft mouse models 226. Nowadays, various combinations of CAR constructs based on antigen-binding receptors and cytosolic domains have been evaluated preclinically for their phagocytic and immuno-stimulating capacities in macrophages 227. As for the CRISPR-Cas9 approach, arginine nanoparticles (ArgNPs) have been used to deliver CRISPR-Cas9 into macrophages to knockout SIRP-α, and this increased their capacity to phagocytose the U2OS osteosarcoma cells 4-fold 228.

One of the biggest challenges for genetically engineered macrophages is the lack of expansion *in vitro* and self-renewal *in vivo* following adoptive transfer. Moreover, macrophages cannot react to HLA and are less effective than CAR-T cells at direct target cell killing. Genetic changes in HSC may lead to off-target effects, such as leukemia or lymphoma. New technologies to expand macrophages, or to identify specific tumor antigens or immuno-stimulatory targets, will have to be implemented in the future.

## 6. Concluding Remarks and Perspectives

In recent years, tumor immunotherapy has seen significant progress. However, patient responsiveness varies significantly across different tumor types and among individuals, and the TME plays a pivotal role in the response to immunotherapy. TAMs represent one of the predominant immune cell types within the TME and interact with their surroundings to influence the immune outcomes. It is essential to elucidate the precise regulatory mechanisms and identify specific targets of TAMs to enhance the efficacy of current immunotherapies. Current research on TAMs continues to face numerous challenges: 1) there is a need for a unified and more scientific approach to identify TAM subtypes; 2) investigations into the mechanisms underlying the interactions between TAMs and the TME remain insufficient; 3) the clinical responses and adverse events associated with TAM-targeted therapies require further evaluation. It is important to note that there may be intrinsic connections among the aforementioned challenges. Despite these limitations, it is encouraging to note that research on TAMs has made significant strides in recent years. For example, single-cell sequencing technologies have provided an opportunity to identify subtypes of TAMs. The metabolic pathways of TAMs have emerged as a novel perspective for subtype identification and as new targets for reprogramming phenotypes. Targeting TAMs, either as a monotherapy or as an adjuvant to chemotherapy and targeted therapies, has received positive feedback from clinical studies. In summary, immunotherapy centered on TAMs is experiencing robust development. The ultimate goal is to reverse the immunosuppressive TME by targeting TAMs, thereby enhancing the efficacy of immunotherapy and ultimately benefiting cancer patients.

## Figures and Tables

**Table 1:** Clinical trials of anti-tumor agents targeting TAMs.

Agent	Target	NCT/UMIN	Phase	Tumor type	Combine with other medicine/therapy	Result	Ref.
Carlumab (CNTO 888)	CCL2	NCT00537368	Phase 1	Solid tumors		Well tolerated, transient free CCL2 suppression, preliminary antitumor activity	150
		NCT01204996	Phase 1b	Solid tumors	Chemotherapy	Well tolerated, neither long-term serum CCL2 suppression nor significant tumor responses	151
Propagermanium	CCL2	UMIN000022494	Phase 1	Breast cancer		Low-grade adverse events, decreased serum IL-6	152
PF-04136309	CCR2	NCT02732938	Phase 1b	PDAC	Gemcitabine and nab-paclitaxel	Increased pulmonary toxicity, no combination therapy advantage	153
CCX872-B	CCR2	NCT02345408	Phase 1b	Pancreatic cancer	FOLFIRINOX	18-month OS rate: 29% for all subjects. no safety issues observed	154
Maraviroc	CCR5	NCT03274804	Phase 1	Colorectal cancer	Pembrolizumab	ORR 5.3%, median PFS 2.1 months, median OS 9.83 months	155
Emactuzumab (RG7155)	CSF1R	NCT01494688	Phase 1a/b	Solid tumors	Paclitaxel	Reduced immunosuppressive TAMs, no antitumor efficacy	156
		NCT02323191	Phase 1b	Solid tumors	Atezolizumab	Elevated ORR, enhanced CD8^+^ T-cell infiltration	157
Lacnotuzumab (MCS110)	CSF1	NCT02435680	Phase 2	Breast cancer	Gemcitabine and Carboplatin	No combination therapy advantage	158
Plerixafor (AMD3100)	CXCR4	NCT00943943	Phase 1	FLT3-ITD-mutated AML	Sorafenib and G-CSF	Improved disease response rate	159
BL-8040	CXCR4	NCT01838395	Phase 2a	AML	Cytarabine	Well tolerated, clinical responses observed	160
Olaptesed pegol (NOX-A12)	CXCL12	NCT01486797	Phase 2a	CLL	Bendamustine and rituximab	Well tolerated, ORR 86%, the median PFS 15.4 months	161
Zoledronate		NCT02181101	Phase 3	Breast cancer	Chemotherapy	No prognosis benefit from prolonged adjuvant zoledronate (> 2 years)	162
Magrolimab (Hu5F9-G4)	CD47	NCT04892446	Phase 2	Multiple myeloma	Daratumumab, pomalidomide/dexamethasone, carfilzomib/dexamethasone, or bortezomib/dexamethasone	NA	163
		NCT02953509	Phase 1b	Non-Hodgkin's lymphoma	Rituximab	Promising therapeutic effect with no significant safety concerns	164
		NCT03869190	Phase 1b/2	Urothelial carcinoma	Atezolizumab	Tolerable, no additive benefit in ORR, PFS, or OS with the addition of magrolimab to atezolizumab	165
CC-90002	CD47	NCT02641002	Phase 1	AML and MDS		No objective responses with CC-90002 monotherapy	166
TTI-621	CD47	NCT02663518	Phase 1	Hematologic malignancies	Rituximab or nivolumab	Well tolerated, therapeutic activity in TTI-621 monotherapy or in combination with rituximab	167
CP-870893	CD40	NA	Phase 1	Solid tumors		Well tolerated with induced objective responses and antitumor activity	168
		NCT00607048	Phase 1	Solid tumors	Paclitaxel and carboplatin	Safe profile, biological/clinical responses observed	169
BCG	TLR2/4	CUETO 93009		Bladder cancer	Mitomycin C	Reduced disease relapse, higher toxicity profile	170
852A	TLR7	NCT0018933	Phase 2	Melanoma		Well tolerated, prolonged stable disease in stage IV metastatic patients	171
		NA	Phase 2	Hematologic malignancies		Safe dosing up to 1.2 mg/m^2^ twice weekly, sustained tolerability/clinical activity	172
Imiquimod	TLR7/8	NCT00899574	Phase 2	Breast cancer skin metastases		Well tolerated with 20% partial response in patients	173
Eganelisib (IPI-549)	PI3Kγ	NCT02637531	Phase 1/1b	Solid tumors	Nivolumab	Antitumor activity in combination group	174
Navoximod (GDC-0919)	IDO1	NCT02048709	Phase 1a	Solid tumors		Favorable tolerability with significant therapeutic efficacy	175
PY314	TREM2	NCT04691375	Phase 1b	Renal cell carcinoma	Pembrolizumab	Limited antitumor efficacy	176
Celecoxib	COX-2	NCT03926338	Phase 2	Colorectal cancer	Toripalimab	Therapeutic efficacy, a high pathological complete response rate and an acceptable safety profile	177
BI 836880	ANG2-VEGF	NCT03972150	Phase 1	Solid tumors	Ezabenlimab	Manageable safety profile with preliminary clinical activity	178
Canakinumab	IL-1β	NCT03631199	Phase 3	NSCLC	Pembrolizumab and platinum-based chemotherapy	No PFS/OS benefit with combination therapy	179
2-DG	Glycolysis	NCT00096707	Phase 1	Solid tumors	Docetaxel	Well tolerated for 63 mg/kg/day 2-DG and docetaxel	180
Metformin	Mitochondrial respiratory chain complex I	NCT01243385	Phase 2	Prostate cancer		Tolerable in non-diabetics, objective prostate-specific antigen responses	181
		UMIN 000002210	Phase 2	Endometrial cancer	MPA	Disease relapse suppression	182
Epacadostat	IDO1	NCT03414229	Phase 2	Sarcoma	Pembrolizumab	Favorable tolerability, limited anti-tumor activity	183
		NCT02752074	Phase 3	Melanoma	Pembrolizumab	Unimproved PFS and OS	184
Tefinostat (CHR-2845)	HDAC	NCT00820508	Phase 1	Hematologic malignancies		Monocyte-selective histone deacetylase inhibition, well tolerated, no dose-limiting toxicities observed	185

PDAC: pancreatic ductal adenocarcinoma; FOLFIRINOX: 5-FU, leucovorin, irinotecan and oxaliplatin; OS: overall survival; ORR: objective response rate; PFS: progression free survival; AML: acute myeloid leukemia; CLL: chromic lymphocytic leukemia; NA: not available; MDS: myelodysplastic syndrome; MPA: medroxyprogesterone acetate; HDAC: histone deacetylase.

## Abbreviations

ICB: immune checkpoint blockade
CAR: chimeric antigen receptor
TME: tumor microenvironment
CTL: cytotoxic T lymphocyte
NK: natural killer
Treg: regulatory T
MDSC: myeloid-derived suppressor cell
TAM: tumor-associated macrophage
M2 TAM: alternatively activated type 2 TAM
TRM: tissue-resident macrophage
CAF: cancer-associated fibroblast
ECM: extracellular matrix
TNBC: triple-negative breast cancer
SDF-1: stromal cell-derived factor-1
M1 TAM: classically activated type 1 TAM
MAM: metastasis-associated macrophage
CSF1: colony-stimulating factor 1
OXPHOS: oxidative phosphorylation
IL-4Rα: IL-4 receptor α
LPS: lipopolysaccharide
HIF-1α: hypoxia inducible factor-1α
TLR: Toll-like receptor
ADCC: antibody-dependent cell-mediated cytotoxicity
iNOS: inducible nitric oxide synthetase
G6P: glucose-6-phosphate
PPP: pentose phosphate pathway
NADPH: nicotinamide adenine dinucleotide phosphate
NOX: NADPH oxidase
DH: isocitrate dehydrogenase
ACOD1: aconitate decarboxylase 1
ITA: itaconate
SDH: succinate dehydrogenase
NO: nitric oxide
2-DG: 2-Deoxy-d-glucose
Arg-1: arginase-1
FAO: fatty acid oxidation
LAL: lysosomal acid lipase
LAM: lipid-associated macrophage
NSCLC: non-small cell lung cancer
GS: glutamine synthetase
GLUL: glutamate-ammonia ligase
α-KG: α-ketoglutarate
GLUD1: glutamate dehydrogenase 1
IDO: indoleamine 2,3-dioxygenase
DC: dendritic cell
EV: extracellular vesicle
HCC: hepatocellular carcinoma
ADAM15: a disintegrin and metalloproteinase 15
ApoE: apolipoprotein E
HLF: hepatocyte leukemia factor
NEDD4-1: neural precursor cell expressed developmentally downregulated protein 4-1
CSC: cancer stem cell
GPNMB: glycoprotein myosone
PDPK1: phosphoinositide-dependent protein kinase 1
PGK1: phosphoglycerate kinase 1
HISLA: HIF-1α-stabilizing long noncoding RNA
TDE: tumor-derived exosome
TCR: T cell receptor
MHC: major histocompatibility complex
IL-15Rc: IL-15Rα complex
Stereo-seq: SpaTial Enhanced REsolution Omics-sequencing
TLS: tertiary lymphoid structure
Breg: regulatory B
cAMP: cyclic adenosine monophosphate
ILC: innate lymphoid cell
LTi: lymphoid tissue-inducer
M-MDSC: monocytic MDSC
PMN-MDSC: polymorphonuclear MDSC
NET: neutrophil extracellular trap
TAN: tumor-associated neutrophil
N1 TAN: anti-tumor TAN
N2 TAN: pro-tumor TAN
CHI3L1: chitinase-3-like-protein-1
α-SMA: α-smooth muscle actin
FSP1: fibroblast-specific protein 1
FAP: fibroblast activation protein
PDGFR: platelet-derived growth factor receptor
MMT: macrophage-myofibroblast transition
EC: endothelial cell
VCAM-1: vascular cellular adhesion molecule-1
PDAC: pancreatic ductal adenocarcinoma
FOLFIRINOX: 5-FU, leucovorin, irinotecan and oxaliplatin
OS: overall survival
ORR: objective response rate
PFS: progression free survival
AML: acute myeloid leukemia
CLL: chromic lymphocytic leukemia
MDS: myelodysplastic syndrome
MPA: medroxyprogesterone acetate
HDAC: histone deacetylase
FPP: farnesyl diphosphate
TRAIL: TNF-related apoptosis-inducing ligand
FRβ: folate receptor β
SIRPα: signal-regulated protein α
LILRB1: leukocyte immunoglobulin-like receptor subfamily B member 1
PI3Kγ: phosphatidylinositol-3-kinase-γ
CRS: cytokine release syndrome
ICD: immunogenic cell death
PGE2: prostaglandin E2
CPT1A: carnitine palmitoyltransferase 1A
TDO: tryptophan 2,3-dioxygenase
HSC: hematopoietic stem cell
VPX: viral protein X
HER2: human EGF receptor 2
ArgNP: arginine nanoparticle
